# Expression of Transposable Elements in Neural Tissues during *Xenopus* Development

**DOI:** 10.1371/journal.pone.0022569

**Published:** 2011-07-26

**Authors:** Fernando Faunes, Natalia Sanchez, Mauricio Moreno, Gonzalo H. Olivares, Dasfne Lee-Liu, Leonardo Almonacid, Alex W. Slater, Tomas Norambuena, Ryan J. Taft, John S. Mattick, Francisco Melo, Juan Larrain

**Affiliations:** 1 Center for Aging and Regeneration and Millennium Nucleus in Regenerative Biology, Pontificia Universidad Católica de Chile, Alameda, Santiago, Chile; 2 Molecular Bioinformatics Laboratory, Millennium Institute on Immunology and Immunotheraphy, Pontificia Universidad Católica de Chile, Alameda, Santiago, Chile; 3 Departamento de Genética Molecular y Microbiología, Facultad de Ciencias Biológicas, Pontificia Universidad Católica de Chile, Alameda, Santiago, Chile; 4 Faculty of Chemical and Pharmaceutical Sciences, Universidad de Chile, Santiago, Chile; 5 Institute for Molecular Bioscience, The University of Queensland, St. Lucia, Queensland, Australia; Radboud University Nijmegen, Netherlands

## Abstract

Transposable elements comprise a large proportion of animal genomes. Transposons can have detrimental effects on genome stability but also offer positive roles for genome evolution and gene expression regulation. Proper balance of the positive and deleterious effects of transposons is crucial for cell homeostasis and requires a mechanism that tightly regulates their expression. Herein we describe the expression of DNA transposons of the *Tc*1/mariner superfamily during *Xenopus* development. Sense and antisense transcripts containing complete *Tc1-2_Xt* were detected in *Xenopus* embryos. Both transcripts were found in zygotic stages and were mainly localized in Spemann's organizer and neural tissues. In addition, the *Tc*1-like elements *Eagle*, *Froggy*, *Jumpy*, *Maya*, *Xeminos* and *TXr* were also expressed in zygotic stages but not oocytes in *X. tropicalis*. Interestingly, although *Tc1-2_Xt* transcripts were not detected in *Xenopus laevis* embryos, transcripts from other two *Tc*1-like elements (*TXr* and *TXz*) presented a similar temporal and spatial pattern during *X. laevis* development. Deep sequencing analysis of *Xenopus tropicalis* gastrulae showed that PIWI-interacting RNAs (piRNAs) are specifically derived from several *Tc*1-like elements. The localized expression of *Tc*1-like elements in neural tissues suggests that they could play a role during the development of the *Xenopus* nervous system.

## Introduction

The complexity of genomes and particularly their transcriptomes has been recognized [1]. Transposable elements are mobile genetic elements that invade new genomes, increasing their copy number and accumulating frame-shift mutations that result in inactive copies [2]. Transposable elements comprise a large proportion of animal genomes, for example 20% in *D. melanogaster*, 36% in *X. tropicalis* and 45% in humans [3], [4]. They are classified into two classes according to their mode of transposition [5]. Class I elements correspond to retrotransposons, which transpose through an RNA intermediate. Class II elements are DNA transposons and transpose through a “cut and paste” mechanism. The relative amount of retrotransposons and DNA transposons varies in different species. Retrotransposons comprise 80% of elements in *D. melanogaster* and 90% in humans [6]. In contrast, 72% of all transposable elements are DNA transposons in *X. tropicalis* [3].

Host cells have developed different mechanisms to silence active transposons and avoid their deleterious effects on genome stability. One strategy, used mainly in the germline, involves the generation of small-interfering RNAs (siRNAs) and PIWI-interacting RNAs (piRNAs) derived from transposable elements that can abolish transposon expression at transcriptional or post-transcriptional levels [7], [8]. siRNAs are generated from double-stranded RNA precursors using the RNAi machinery. In contrast, a ping-pong model dependent on PIWI-proteins has been proposed for piRNAs biogenesis [9], [10].

While transposons have been regarded as having a negative role in cellular processes, recent evidence suggests that transposons can also have positive role, including the regulation of gene expression by regulating the chromatin conformation [11], [12], the addition of novel regulatory elements into gene networks [13], the formation of novel proteins or protein domains [14] and the generation of neuronal variability by transposition to different genome positions during neural development [15], [16]. The differential expression of several retrotransposon families in *Drosophila* and mouse embryos suggests that they play a role in embryonic development [17], [18], [19].

In a previous analysis we described novel transcripts with differential expression along the dorso-ventral axis at the gastrula stage of *X. tropicalis* [20]. One of these transcripts corresponds to a DNA transposon expressed specifically in the Spemann's organizer, a tissue that is required for proper dorso-ventral and anterior-posterior patterning of the embryo [21]. This novel DNA transposon belongs to the superfamily of *Tc*1-like elements widely distributed among animal genomes [3], [22], [23]. Transcripts for some of these *Tc*1-like elements have been detected in EST databases but no studies on how their expression is regulated or about their possible function have yet been described [24], [25], [26], [27], [28], [29], [30].

DNA transposons are the most abundant class in the *X. tropicalis* genome [3]. Seven lineages of *Tc*1-like elements have been described: *Eagle*, *Froggy*, *Jumpy*, *Maya*, *Xeminos*, *TXr* and *TXz* [22]. In *X. laevis*, two *Tc*1-like elements have been described, *TXr* and *TXz* [31]. Although the expression of *TXr* has been described in *X. laevis* in endodermal tissues at later stages [24], a detailed analysis of the expression of these elements during *Xenopus* development has not been previously performed.

Here we describe the regulated expression of DNA transposons from the *Tc*1/mariner superfamily. Sense and antisense transcripts of *Tc1-2_Xt* can be detected at higher levels after mid-blastula transition (MBT). At gastrula stage it is highly enriched in dorsal tissues and is subsequently localized primarily to neural derivatives. In addition, the zygotic expression of several *Tc*1-like elements was observed during *X. tropicalis* development. Interestingly, the *Tc1-2_Xt* transcript was not detected in *X. laevis* embryos. However, a similar expression pattern was found for two *Tc*1-like elements during *X. laevis* development. Importantly, deep sequencing analysis demonstrated that piRNAs derived from these primary transcripts are present during *Xenopus* development. In the case of *Tc1-2_Xt*, RT-PCR and Northern Blot validated the expression of its specific piRNAs. This is the first detailed characterization of the temporal and spatial regulated expression of *Tc*1-like elements during *Xenopus* development.

## Results

### Characterization of *Tc1-2_Xt* in the *X. tropicalis* genome and its expression during development

**Figure 1:**
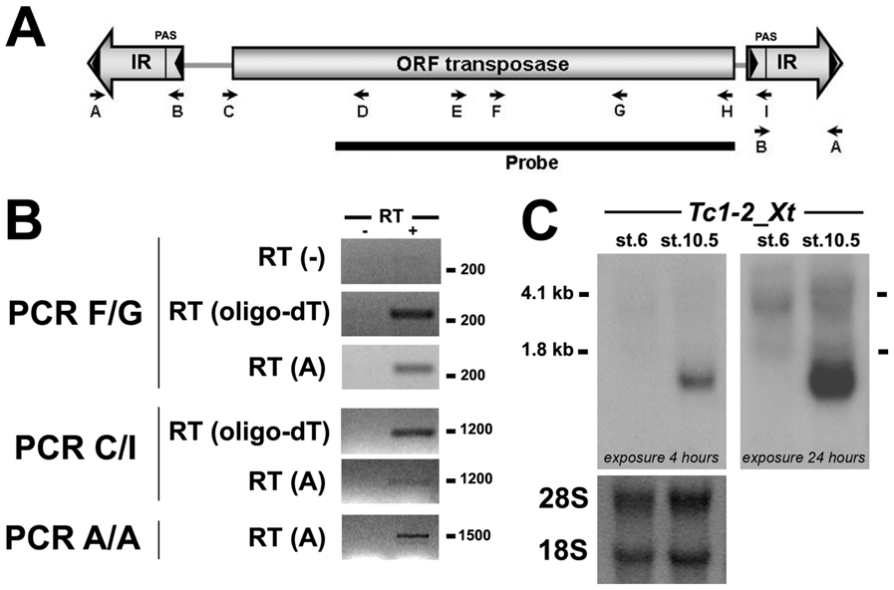
Characterization of a novel *Tc*1-like element in *X. tropicalis*. **A**) Scheme of the *Tc1-2_Xt* transposable element showing ORF, inverted repeats (IRs), direct repeats (black triangles inside the IRs), the polyadenylation signal (PAS), primers (arrows) and the probe used in this study for Nortehrn Blot and *in situ* hybridization analysis. **B**) RT-PCR with total RNA from gastrula stage using different primers. Reactions without (−RT) or with reverse transcriptase (+RT) are included. **C**) Northern blot using the probe shown in A for 10 µg of total RNA of stage 6 and stage 10.5 *X. tropicalis* embryos. Two different exposures are shown. Ribosomal RNAs are shown as loading controls.

A previous screen showed that a fragment of a DNA transposon was expressed specifically in the dorsal region at the gastrula stage of *X. tropicalis* [20]. We decided to perform a more in-depth analysis of this element. We searched this element in RepeatMasker and Repbase databases [32], [33]. This element is identified as *Tc1-2_Xt* in RepeatMasker database but it was not found in Repbase. It is important to mention that Repbase contains an element identified as *Tc1-2_Xt* but it corresponds to a different element, identified as *Tc1DR3* in RepeatMasker and as *maya* according to Sinzelle et al [22]. Therefore, we used the name *Tc1-2_Xt* for the novel element identified in this work according to the RepeatMasker nomenclature and maintained *maya* to the previous identified element to avoid confusion. *Tc1-2_Xt* is distant to other described *Tc*1-like elements in *X. tropicalis* [22] and is therefore the founding member of a new family of *Tc*1-like elements (Figure S1 and File S1). The *Tc1-2_Xt* family has an average length of 1,581 bp and contains the typical structure of DNA transposon organization; the transposase ORF is flanked by two 199 bp Inverted Repeats (IRs) which include two 17 bp direct repeats (DRs) (Figure 1A). In the *X. tropicalis* genome sequence, 116 highly similar copies (>93% sequence identity among them) of a complete *Tc1-2_Xt* were identified. Detailed sequence analysis indicated that only 5 out of the 116 genomic copies encode a potentially functional transposase. BLAST searches retrieved related sequences only in *Danio rerio* (zebrafish) and *Gasterosteus aculeatus* (stickleback) genomes and further analysis determined that these sequences were distantly related to *Tc*1 elements found in other genomes (Figure S2 and File S2).

To characterize the *Tc1-2_Xt* transcript, we performed RT-PCR and Northern blot analyses from gastrula stage of *X. tropicalis*. RT was performed using a specific primer for the 5′-end of the IR (primer A), followed by PCR amplification with specific primers for the transposase ORF (F and G, C and I primer pairs) or the IR (primer A and B) obtaining fragments of the expected sizes (Figure 1A, B). No amplification product was detected in the absence of reverse transcription (without reverse transcription) indicating that the product is specifically obtained from cDNA and it is not derived from genomic contamination. The fact that the PCR amplification with primer A yields a fragment of 1500 bp suggested that the complete transposon is transcribed. Identical results were obtained when the RT reaction was performed using an oligo-dT primer, indicating that these RNAs are poly-adenylated. The detection of PCR fragments derived from reverse transcription using the primer A and oligo-dT indicates that the population of RNAs is mainly composed from both complete elements and polyadenilated transcripts. All cDNAs sequenced (23 out of 23 clones) containing the putative ORF (fragment C/I) presented mutations and frame-shifts and none of them could encode for an active transposase (data not shown). Northern blot analysis yielded a single and specific band below ∼1.8 kb demonstrating that the main transcript present at the gastrula stage corresponds to the complete transposon (Figure 1C). It is noteworthy that this band is sharp, suggesting that the majority of the transposon RNA is homogenous and not derived from chimeric transcripts with cellular genes as reported in other studies [19]. In addition, this result suggests that the sequence recognized by the cDNA probe used in the Northern Blot is not present in transcripts of different lengths. Only longer exposure revealed some higher molecular weight smearing that could correspond to a small fraction of chimeric RNAs (Figure 1C). We concluded that most of the *Tc1-2_Xt* transcripts expressed at the gastrula stage contained a full-length version of the transposon. Because the individual copies of this element were nearly identical, we were unable to identify the precise genomic locus(loci) that is(are) responsible for the expression of *Tc1-2_Xt*.

### Differential expression of *Tc1-2_Xt* during *X. tropicalis* development

**Figure 2:**
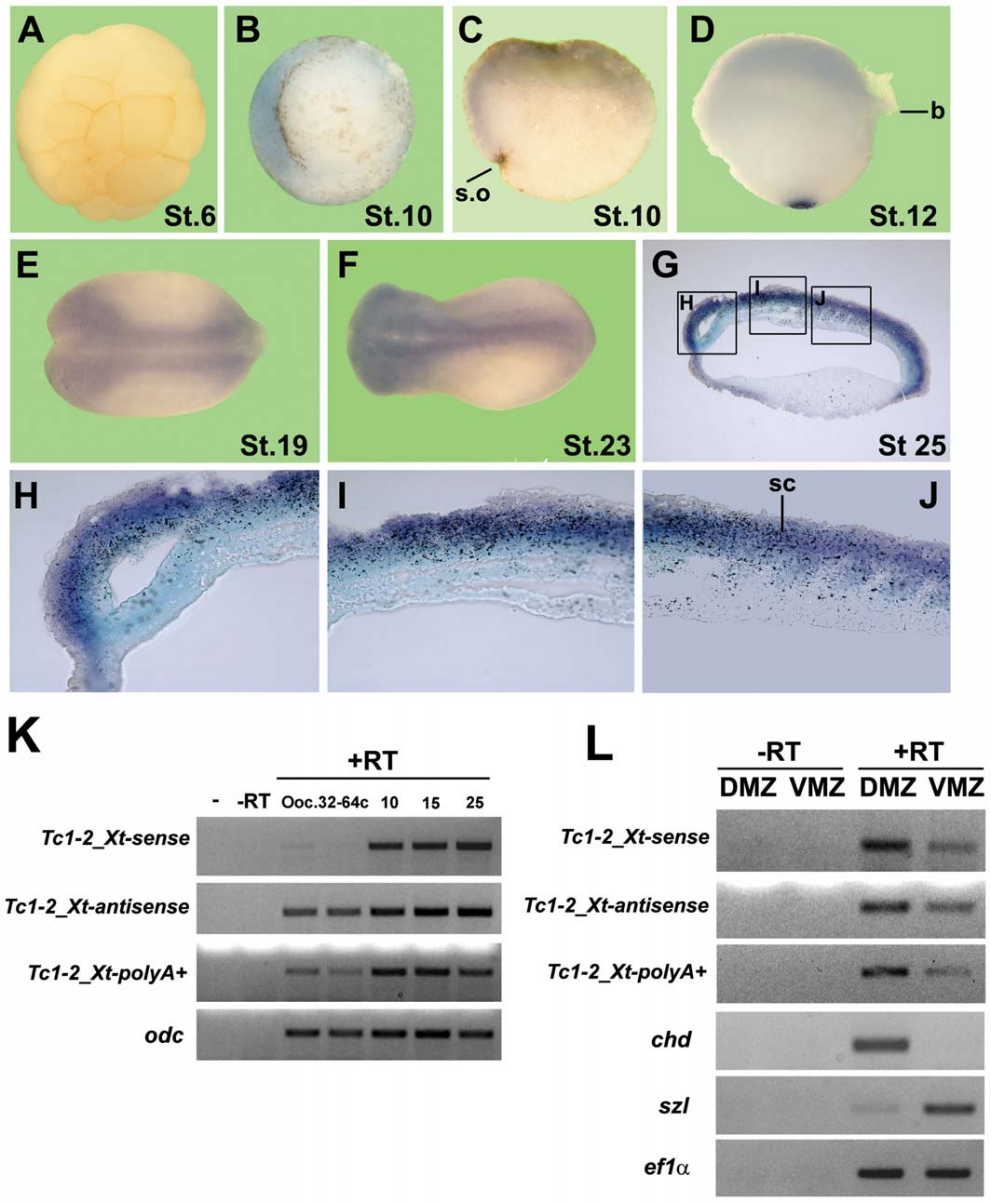
*Tc1-2_Xt* regulated expression during *X. tropicalis* development. *In situ* hybridization with a specific sense probe for *Tc1-2_Xt* in embryos at stage 6 (**A**), 10 (**B**), 12 (**D**), 19 (**E**) and 23 (**F**). Hemi-section of a gastrula stage embryo is included (**C**). Sagital section of a stage 25 embryo (**G**) showing the staining at the dorsal side (internal layer). Three images at higher magnifications are included (**H**, **I**, **J**). **K**) Reverse transcription was performed using primer H for the sense and C for the antisense strand (see Figure 1A) and oligo-dT for the polyA+ fraction of *Tc1-2_Xt* in different stages of *X. tropicalis* development. PCR was performed using F/G primers (see Figure 1A). A PCR reaction without cDNA is included as control (-); *odc*, ornithine decarboxylase, *Chd*, chordin; *szl*, sizzled; *ef1*α, elongation factor 1a. **L**) RT-PCR for dorsal and ventral explants of *X. tropicalis* gastrula (stage 10). Reactions with (+RT) or without (-RT) reverse transcriptase are included. s.o, Spemanńs organizer; b, blastopore; sc, spinal cord.

We have previously reported that the sense strand of *Tc1-2_Xt* is differentially expressed at gastrula stage [20]. Since expression of both strands of *Tc*1 has been previously described in *C. elegans* [29], we performed a more detailed analysis of the expression of both strands during *X. tropicalis* development. *In situ* hybridization analysis showed that sense and antisense transcripts of *Tc1-2_Xt* were weakly detected at maternal stages and increased at gastrula stage (Figure 2A, 2B and Figure S3). At gastrula stage, both transcripts were highly enriched in the Spemann's organizer (Figure 2B, 2C and Figure S3B) and were localized mainly at the dorsal side at neurula stages (Figure 2B and Figure S3C,D). At later stages, *Tc1-2_Xt* expression was observed in neural tissues, especially in the prospective brain and spinal cord (Figure 2E–J and Figure S3E-H). Consistently, RT-PCR analysis of the poly-adenylated fraction showed higher levels of *Tc1-2_Xt* transcripts after mid-blastula transition (Figure 2K, compare stages 10, 15 and 25 with oocytes and embryos with 32–64 cells). Interestingly, sense transcripts were not detected in oocytes but important levels of the antisense strand were detected (Figure 2K). RT-PCR showed that both strands were enriched dorsally at gastrula stage (Figure 2L) giving further support to *in situ* hybridization results. In summary, *Tc1-2_Xt* sense and antisense transcripts levels are temporally and spatially regulated during *X. tropicalis* development. The fact that the levels of *Tc1-2_Xt* mRNA are regulated gives further support to the specificity of the PCR fragments amplified.

Interestingly, *Tc1-2_Xt* transcripts were not detected in maternal or gastrula total RNA samples of *X. laevis* by Northern Blot and RT-PCR analysis (data not shown). PCR using *X. laevis* genomic DNA with specific primers for *Tc1-2_Xt* did not produce any amplification. These results suggest that *Tc1-2_Xt* is present only in the *X. tropicalis* genome.

### Expression of other *Tc*1-like elements during *Xenopus* development

**Figure 3:**
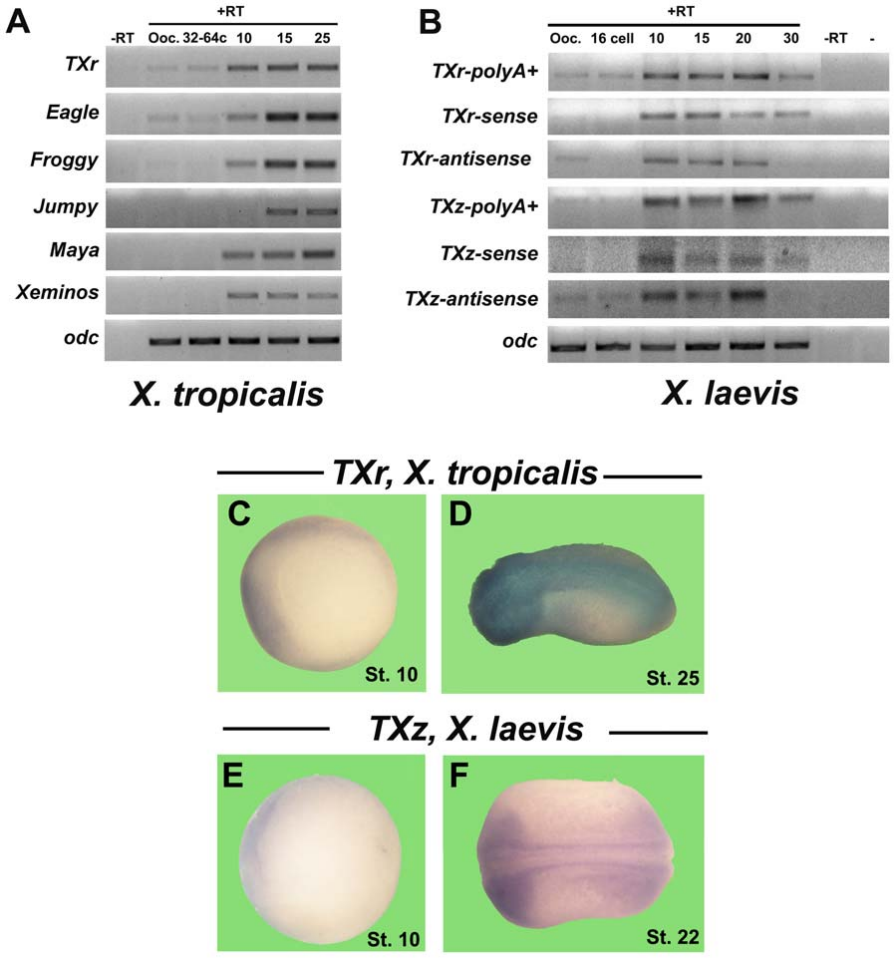
Zygotic expression of *Tc*1-like elements during *Xenopus* development. **A)** RT-PCR of the poly-adenylated fraction of *Tc*1-like elements in different stages of *X. tropicalis* development. **B)** RT-PCR for the poly-adenylated fraction (polyA+), the sense and antisense strands of *TXr* and *TXz* in different stages of *X. laevis* development. Reactions with (+RT) or without (-RT, stage 10) reverse transcriptase are included. Ooc, oocytes. *In situ* hybridization in *X. tropicalis* embryos using an antisense probe to specifically detect the sense strand of *TXr* at stage 10 **(C)** and 25 **(D)**. *In situ* hybridization in *X. laevis* embryos using a sense probe to specifically detect the antisense strand of *TXz* at stage 10 **(E)** and 22 **(F)**.

In the *X. tropicalis* genome, several DNA transposon families have been described [22]. However, to our knowledge there are no studies showing their transcription. To determine if some of these *Tc*1-like elements are also expressed during *X. tropicalis* development, RT-PCR using oligo-dT to detect poly-adenylated transcripts was performed in total RNA from oocytes and different stages of development. Transcripts for all the *Tc*1-like elements studied (*TXr*, *Eagle*, *Froggy*, *Jumpy*, *Maya* and *Xeminos*) were specifically detected and expressed mainly at zygotic stages in *X. tropicalis* (Figure 3A). These results indicate that the levels of *Tc*1-like transcripts are regulated during *X. tropicalis* development.

In *X. laevis*, two *Tc*1-like elements have been identified in the genome [31]. To determine if these elements are also expressed, RT-PCR using oligo-dT to detect poly-adenylated transcripts was performed. Poly-adenylated transcripts of *TXr* and *TXz* were detected mainly at zygotic stages of development (Figure 3B). Considering that expression of both strands was demonstrated for *Tc1-2_Xt* in *X. tropicalis*, we performed specific strand RT-PCR for these elements. Both strands of *TXr* and *TXz* are expressed during *X. laevis* development (Figure 3B). *TXr* and *TXz* sense and antisense transcripts are predominantly detected after the beginning of the zygotic transcription. Therefore, similarly to *X. tropicalis Tc*1-like elements, *TXr* and *TXz* are expressed during *X. laevis* development.

*TXr* expression has been described in liver at 41–44 stages in *X. laevis* by *in situ* hybridization [24]. To characterize the spatial pattern of expression for both strands of *TXr* and *TXz* at earlier stages of *X. tropicalis* and *laevis* development, *in situ* hybridization was performed. Sense and antisense transcripts for *TXr* and *TXz* are enriched in the dorsal side at gastrula (Figure 3C, E and Figures S4B and S5B). At later stages they are preferentially enriched in dorsal and neural tissues (Figure 3D, F and Figures S4C-F, S5C-F and S6B-D, G-J). In summary, we demonstrated that two *Tc*1-like elements (*Tc1-2Xt* and *TXr*) in *X. tropicalis* and two in *X. laevis* (*TXr* and *TXz*) are differentially expressed during development.

### Identification and expression of small RNAs derived from *Tc*1-like elements

**Figure 4:**
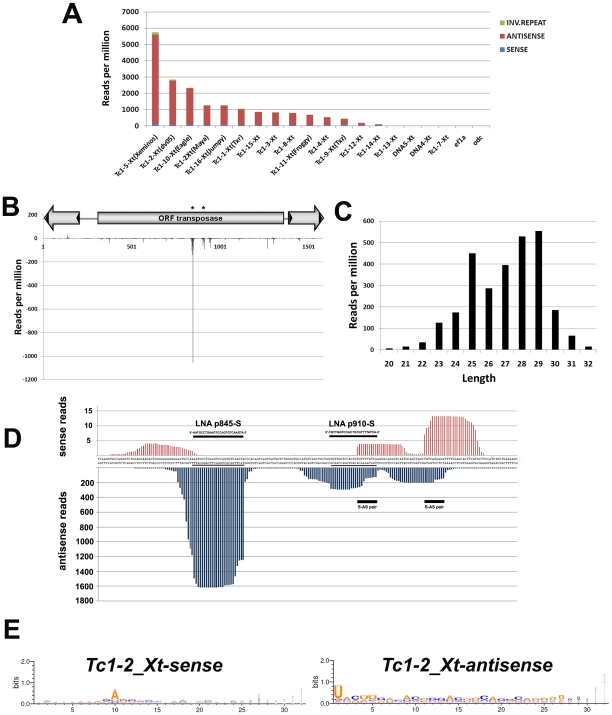
Small RNAs derived from *Tc*1-like elements and detailed analysis of *Tc1-2_Xt*-specifically derived piRNAs. **A)** Reads per million mapped to several *Tc*1-like elements at the gastrula stage. Reads mapped to *ef1α* and *odc* are included. Only reads mapped specifically to a single *Tc*1-like element were considered. **B**) Scheme of the reads mapped to *Tc1-2_Xt*, showing two specific points (*p845* and *p910*, asterisks). The Y-axis corresponds to the sum of reads for each nucleotide position, considering only the 5′-end of small RNAs. **C)** Histogram of the length of small RNAs mapped to *Tc1-2_Xt*. **D**) Zoom of *Tc1-2_Xt* sequence representing the region containing the more abundant small RNAs detected. The sequences were plotted against the sum of reads obtained for each nucleotide position after mapping all *Tc1-2_Xt* small RNAs. In this case, all positions of small RNAs were considered. LNA sequences and two sense-antisense pairs are shown. Underlined sequences correspond to piRNAs *p845* and *p910*. Scale of Y-axis was scaled to show sense reads. **E)** Weblogos of small RNAs mapped to *Tc1-2_Xt* according to the orientation of the transposon sequence.

**Figure 5:**
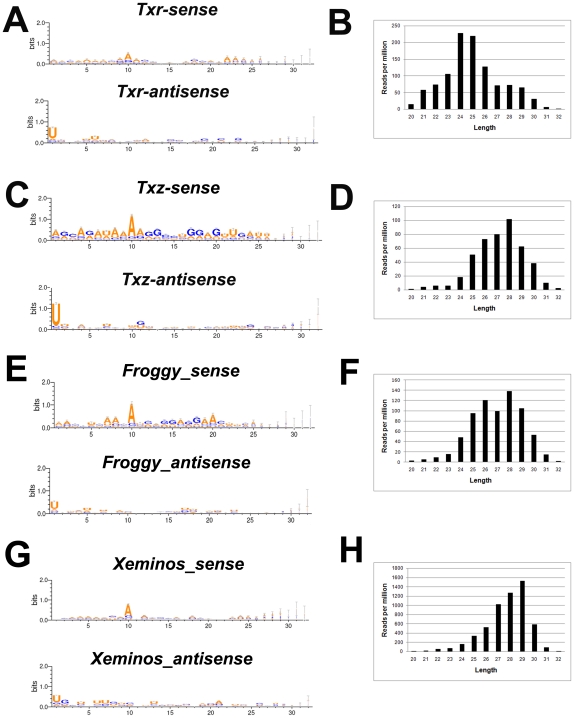
Analysis of piRNAs mapped to *Tc*1-like elements. **(A,C,E,G)** Weblogos of small RNAs mapped to *TXr, TXz, Froggy and Xeminos* according to the orientation of the transposon sequence. **(B,D,F,H)** Histogram of the length of small RNAs mapped to *TXr*, *TXz, Froggy* and *Xeminos.*

Transcripts of transposable elements are a source for the synthesis of endogenous siRNAs and piRNAs [7]. To determine whether small RNAs are derived from *Tc1-2_Xt* and other elements, we performed deep sequencing of small RNAs isolated from explants of gastrula stage *X. tropicalis* embryos. We obtained 17,553,124 reads between 20 and 32 nucleotides with exact match to the genome (See Material and Methods, NCBI accession number GSE30067). A different amount of perfectly matched reads for each *Tc*1-like element was found (Figure 4A and Table S1). These small RNAs were specific and not a consequence of degradation products, because they rarely mapped to abundant transcripts such as *ef1α* and *odc* (Figure 4A). Most reads were antisense relative to the transposon sequences (red color, Figure 4A and Table S1). A detailed analysis of small RNAs derived from *Tc1-2_Xt*, *TXr* and *TXz* was performed (Figures 4 and 5). Most of *Tc1-2_Xt-*derived small RNAs (94.3%) were antisense relative to the transposon sequence and 95% of them between 23 and 30 nucleotides long (Figure 4B, C). For further analysis we focused the region that generated most of *Tc1-2_Xt*-derived small RNAs (*p845* and *p910*) and plotted them as a function of the sum of reads per nucleotide position. We detected the presence of sense-antisense pairs with an overlap of 10 nucleotides, which is a characteristic feature of piRNAs according to the ping-pong biogenesis model (S-AS pair, Figure 4D) [9], [10]. In addition, sense-derived small RNAs exhibited a bias for an adenine base (A) in the 10^th^ position, while antisense small RNAs were biased towards a uracil (U) in the 1^st^ position (Figure 4E). These results strongly suggest that a ping-pong mechanism is involved in the biogenesis of *Tc1-2_Xt*-derived small RNAs and that they are predominantly PIWI-interacting RNAs (piRNAs). Furthermore, 80.9% of the reads that mapped to *Tc1-2_Xt* were found in two databases containing piRNAs from *X. tropicalis* eggs (Table S1) [34], [35]. Similar results for length distribution, bias in the 1^st^ and the 10^th^ position and presence in the published piRNA databases were obtained for *TXr*, *TXz* and other *Tc*1-like elements (Figure 5, Figure S7, Table S1 and data not shown) suggesting that transposon derived piRNA are generated via a ping-pong mechanism. In the case of *TXr*, the length distribution showed a peak in 24–25 nucleotides in contrast to the peak in 27–28 nucleotides for most of *Tc*1 elements. However, this distribution also suggests that these small RNAs are piRNA, consistent with the bias in the 1^st^ and the 10^th^ position and the presence in piRNA libraries.

**Figure 6:**
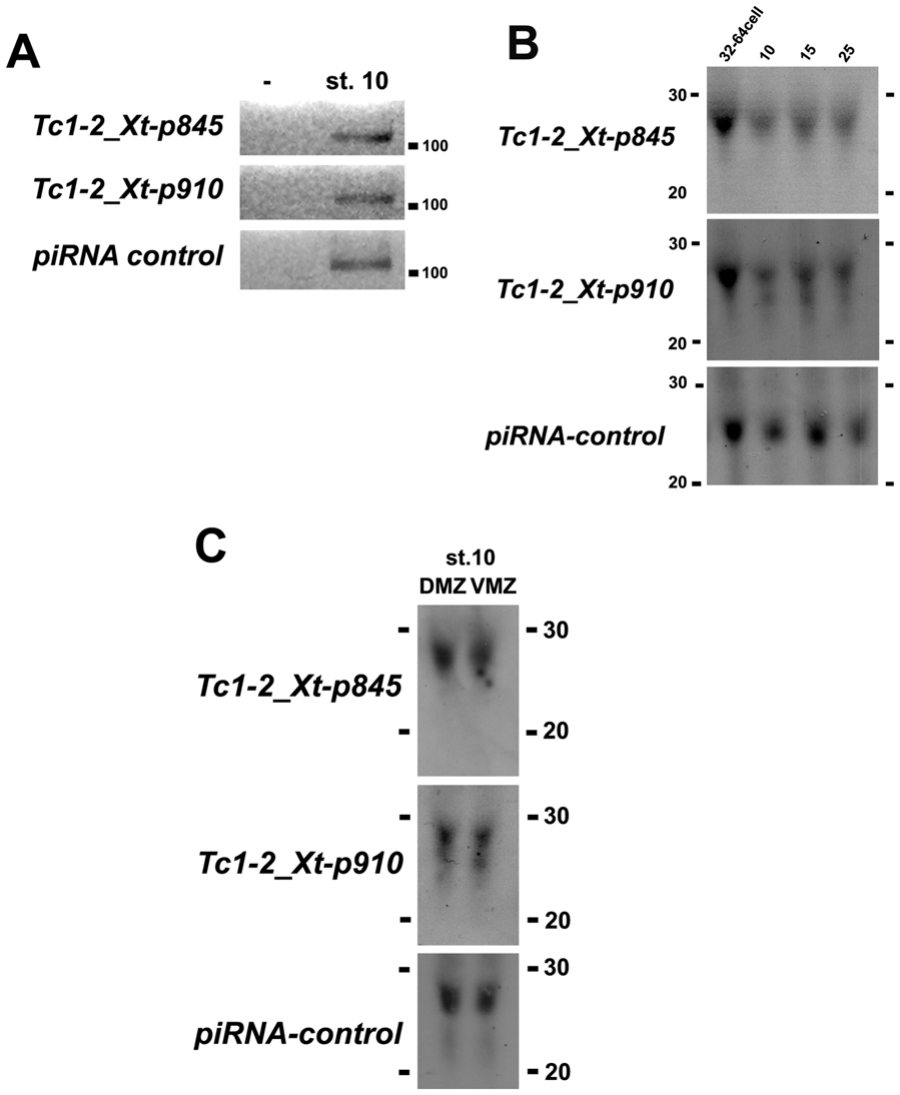
Expression of *Tc1-2_Xt*-derived piRNAs in *X. tropicalis*. **A)** RT-PCR for piRNAs *p845* and *p910* from total RNA of embryos at gastrula stage. A PCR reaction without cDNA is included as control (-). **B)** Northern blot for *p845* and *p910* sequences using 25 µg of total RNA from 32–64 cell, gastrula (stage 10), neurula (stage 15) and tadpole (stage 25). A high expression piRNA was used as a loading control. **C**) Northern blot for *p845* and *p910* sequences using 20 µg of total RNA from dorsal and ventral explants of *X. tropicalis* gastrula.

To validate the results from the deep sequencing experiment, and to study the expression of *Tc1_2Xt*-derived piRNAs two abundant small RNAs (antisense relative to *Tc1-2_Xt*) were selected for further analysis (*p845* and *p910*, Figure 4B, asterisks and Figure 4D, underlined sequences). Bioinformatics analysis showed that these two piRNA sequences are within the multiple genomic transposon regions, supporting our hypothesis that *p845* and *p910* are derived from *Tc1-2_Xt* (data not shown). These piRNAs were amplified at gastrula stage by RT-PCR for small RNAs (Figure 6A) and their identity confirmed by sequencing (data not shown). Northern blots were performed using Locked Nucleic Acids (LNA) complementary to *p845* and *p910* (p845-S and p910-S, Figure 4D) and higher levels of expression of *p845* and *p910* were observed at maternal stages (32-64 cells) compared to stage 15 (Figure 6B). A similar trend during development, although not statistically significant, was observed by qRT-PCR for small RNAs (Figure S8A,B). In contrast to the temporal regulation, no difference was detected in the spatial distribution of *p845* and *p910* using northern blot (Figure 6C) and qRT-PCR analyses of dorsal and ventral explants (Figure S8C). It is noteworthy to mention that the expression levels of *Tc1-2_Xt*-derived piRNAs inversely correlate to those of the full-length transcript during *Xenopus* development (compare Figures 2K and 6B), supporting the hypothesis that piRNAs could be involved in the regulation of *Tc1-2_Xt* expression.

## Discussion

Transposable elements are considered an important source of regulatory sites for gene expression, of novel proteins or protein domains for the host and it has been shown to be involved in the generation of variability during the formation of the nervous system [13], [14], [36]. In addition, their contribution to transcriptomes has recently been recognized [19], [37]. The differential expression of retrotransposons during development has been described mainly in *Drosophila* and mouse [17], [18], [19]. Here we showed that the expression of members of a new family of the *Tc*1 DNA transposon superfamily is regulated temporally and spatially during *Xenopus* development. Although our RT-PCR results do not discard that other cellular transcripts could contain *Tc1-2_Xt* fragments, Northern Blot analysis suggest that this element is preferentially transcribed independently of other genes and does not form chimeric transcripts as described in mouse [19]. Sequence analysis of 23 *Tc1-2_Xt* cDNA copies showed that any of them encode for an active transposase. However this is far from being an exhaustive analysis, especially if we consider that 5 out of 116 genomic copies of the transposon element encode for an active transposase. In the case of *TXr*, several intact copies were previously detected in the first draft of the *X. tropicalis* genome but no intact ORFs for *TXz* were identified and transcription of these elements was not studied [22]. Therefore, for *Tc1-2_Xt* or *TXr*, low transcriptional levels of an active transposase cannot be discarded.

The high sequence identity of the transposon copies in the genome did not allow for determination of the *locus* or *loci* from which it is transcribed. It is not known if these elements contain their own regulatory sites for expression. The presence of promoters in the inverted repeats have only been described in a *Tc*1 element from fungus [38]. In contrast, in *C. elegans*, it was suggested that *Tc*1 transcripts were originated from fortuitous read-through transcription, probably by host promoters [29].

We were not able to detect any ortologues of *Tc1-2_Xt* in the genome or transcriptome of *X. laevis*. There are several explanations for this result. It is likely that the *Tc1-2_Xt* transposon invaded the *X. tropicalis* genome after the divergence between both species, for example, by horizontal transfer. Another possibility is that the sequence of *Tc1-2_Xt* has substantially diverged in the *X. laevis* genome and thus we were not able to detect it through PCR, Northern blot or *in situ* hybridization. Without the sequence of the *X. laevis* genome available it is difficult to distinguish between these alternatives and detailed analyses must be performed to explain the absence of this element in *X. laevis*. Similar analysis can be performed for the other *Tc*1-like elements present in the *X. tropicalis* genome. However, it is very interesting that *TXr* and *TXz* elements (present in both genomes and sharing high sequence identity) are expressed in a similar fashion in both species during development. In addition, their expression patterns, mainly in the neural tissue, are highly similar to the expression of *Tc1-2_Xt* in *X. tropicalis*. These results may suggest that the expression of transposable elements is regulated and potentially is required for the proper formation of the neural tissue. Alternatively, it is also possible that the expression patterns observed are the consequence of integrations near enhancer sequences which promote neural expression.

Although we did not determine if the small RNAs derived from *Tc1-2_Xt* are bound to the XIWI protein, these small RNAs have the typical characteristics of piRNAs: 23 and 30 bp long, most are antisense relative to the transposon sequence and have a bias for uracil in the 1^st^ position and adenine in the 10^th^ position. In addition, an important fraction of these sequences are present in published *Xenopus* piRNA libraries [34], [35], [39]. The same characteristics were observed for the other *Tc*1-like element derived small RNAs. Therefore, these results strongly suggest that these small RNAs belong to the piRNA class.

*Tc1-2_Xt*-derived piRNA levels are higher in oocytes, when the levels of *Tc1-2_Xt* transcripts are less abundant, while the opposite holds true for the zygotic stage. These results suggest that piRNAs could regulate the levels of the DNA transposon transcripts at early stages of development possibly to avoid the deleterious consequences of transposition in the germline and/or to control their developmental function(s). One possible mechanism for temporal regulation of *Tc1-2_Xt* levels is the cleavage of the *Tc1-2_Xt* transcripts by piRNAs. The presence of transcripts from both strands of *Tc1-2_Xt* is consistent with the ping-pong cycle for biogenesis of piRNAs and a possible cleavage of *Tc1-2_Xt* [9], [10]. In addition, it has been described that piRNA can induce the deadenylation of maternal mRNA [40] and a similar mechanism could be involved in the temporal regulation of *Tc1-2_Xt*. One possible approach for studying the effect of piRNAs in the expression of *Tc1-2_Xt* would be the depletion of PIWI proteins in oocytes and/or early development in *X. tropicalis*. However, we were not able to decrease the levels of PIWI proteins by injecting morpholinos in oocytes (data not shown) and therefore we could not address this important question.

Interestingly, the transposon is transcribed after MBT and higher levels are specifically detected in dorsal tissues such as Spemann's organizer and neural tissues. The fact that *Tc1-2_Xt-*derived piRNAs are uniformly expressed at gastrula stage suggests that piRNA can induce cleavage or deadenylation more actively in the ventral side of the embryo, a hypothesis that needs further testing. Alternatively, the differential expression along the dorso-ventral axis of *Tc1-2_Xt* transcripts is not a consequence of piRNAs cleavage or deanylation. It would be interesting to determine the spatial pattern of expression of *Tc1-2_Xt-*derived piRNAs by *in situ hybridization* during development. However, the probes could also detect the *Tc1-2_Xt* transcripts, therefore, the interpretation of these results could be difficult.

piRNAs may not be used solely as a blanket strategy for silencing transposition in the germline, but as a means of regulating transposon expression during development. We found that all genomic matches of the *Tc1-2_Xt*-derived piRNAs *p845* and *p910* correspond to the *Tc1-2_Xt* sequence and therefore, they likely only regulate the levels of *Tc1-2_Xt* and not those of other endogenous transcripts in *X. tropicalis*, as described for other genes in *Drosophila* [40], [41]. However, we cannot discard that other piRNAs derived from this element can regulate other genes. A similar scenario can be proposed for *TXr* or *TXz* in *X. tropicalis* or *X. laevis*.

The dorsal and neural expression of *Tc1-2_Xt*, *TXr* and *TXz* allows speculation about a possible role for these transposable elements in neural development. This tissue-specific transcription of non-active copies can be involved in the generation of a specific chromatin conformation, as described in the mouse pituitary development [12]. Another hypothesis would be that active elements could be involved in the generation of heterogeneity during nervous system development. The Gagés laboratory has demonstrated that endogenous retrotransposition occurs in the vicinity of neural genes during the development of the mouse nervous system. This has been suggested as a novel mechanism involved in the generation of neuronal diversity [15], [16], [42]. Whether active copies of *Tc1-2_Xt*, *TXr* and *TXz* are present in neural tissues or if endogenous transposition occurs in these tissues remain to be determined. At the present, little is known about the biology and action of DNA transposons during early development, and that the current study has shed more light on this important class of mobile elements.

## Materials and Methods

### Bioinformatics analysis

The *X. tropicalis* genome from Joint Genome Institute was used (v4.1). An *in silico* PCR was performed using a 37-nt primer corresponding to the end of the transposon. All against all pairwise alignments of the extracted sequences were performed using ClustalW with default parameters. Single linkage hierarchical clustering of the aligned sequences was produced based on the observed percentage sequence identity of each pairwise alignment. RepeatMasker and Repbase databases were used to search for transposable elements [32], [33]. A comparison between both databases was performed in order to consider the different nomenclatures of some elements. Sequence alignments against several genomes (*Homo sapiens*, *Mus musculus*, *Rattus novergicus*, *Gallus gallus*, *Takifugu rubripes*, *Oryzias latipes*, *Gasterosteus aculeatus*, *Tetraodon nigroviridis*, *Danio rerio*, *Drosophila melanogaster* and *Caenorhabditis elegans*) were performed using BLAST with default parameters.

### Embryo and oocyte manipulation

*X. tropicalis* manipulations were performed as described (http://tropicalis.berkeley.edu/home/index.html). Female *X. tropicalis* were anaesthetized, ovaries surgically removed and Stage V-VI oocytes manually defolliculated. Dorsal (dorsal marginal zone, DMZ) and ventral explants (ventral marginal zone, VMZ) were obtained from early gastrula embryos (vegetal view to see the dorsal lip of the blastopore). *In vitro* fertilizations, embryo culture, explants culture and *in situ* hybridizations of *X. laevis* were performed as described [43]. Probes for *in situ* hybridizations were synthesized from PCR products cloned by using specific primers (Table S2). Manipulations of Xenopus embryos were performed according to the protocols approved by the “Comision de Bioetic y bioseguridad” from the Faculty of Biological Sciences, P. Universidad Catolica de Chile on July 6, 2006.

### RT-PCR

Total RNA from *X. tropicalis* and *X. laevis* embryos or oocytes was isolated using TRIzol reagent and treated with DNase I (Invitrogen). RT-PCR analyses were performed in the linear phase of amplification (25–30 cycles) using primers listed in Table S2.

### Northern blot of *Tc1-2_Xt* mRNA

Total RNA from stage 6 (maternal) and 10 (gastrula) *X. tropicalis* embryos was isolated using TRIzol (Invitrogen) and electrophoresed on agarose gels, transferred to a Hybond-XL membrane (Amersham) and UV-crosslinked. Membranes were blocked with Herring DNA sperm (Invitrogen). A cDNA probe (25 ng) was labelled with [α-^32^P] dCTP using Rediprime II Random Prime Labelling System (Amersham) and hybridization was performed in ULTRAhyb hybridization buffer (Ambion). Membrane was washed with 2X SSC/0.1% SDS, followed by washes with 0.2X SSC/0.1% SDS and exposed for different times. Stripping of membranes was performed as previously described [44].

### RT-PCR for small RNAs

Total RNA (5 ug) was isolated using TRIzol and RT-PCR and qRT-PCR were performed as previously described [45]. Normalization was performed with a piRNA control as previously described [46]. PCR products were purified, cloned and sequenced.

### Northern blot for small RNAs

Northern blots were performed using 15–25 µg of total RNA/lane as previously described [47], [48]. LNA probes for *Tc1-2Xt* and a highly expressed piRNA were labelled with [γ-^32^P]-ATP using T4-kinase (New England Biolabs) and purified with G25 columns (GE Healthcare).

### Deep sequencing of *X. tropicalis* small RNAs and analysis of reads

Total RNA from dorsal and ventral explants at gastrula stage of *X. tropicalis* was isolated by using TRIzol, small RNAs were extracted from gel after electrophoresis and processed for Solexa/Illumina sequencing technology. Briefly, samples were individually prepared and sequenced at GeneWorks (Adelaide, Australia), a commercial sequencing provider. Libraries were generated using the v1.5 Illumina small RNA sequencing kit and adapter (5′ ATCTCGTATGCCGTCTTCTGCTTG 3′) according to the manufacture's instructions. Following sequencing, adaptors were removed using in-house scripts and the FASTX toolkit program FASTQ/A clipper (http://hannonlab.cshl.edu/fastx_toolkit/). Tags that had mis-read bases (e.g. Ns) or were less than 15 nt were excluded to generate a first set. The raw data has been deposited in GEO-NCBI under accession number GSE30067. These two sequencing experiments were combined to establish a complete transcriptional snapshot of the small RNAs in the *Xenopus* gastrula. Then, sequences between 20 and 32 bases were selected. Mapping of small RNAs to the *X. tropicalis* genome sequence and to representative *Tc*1-like sequences was performed using Bowtie, with the constraint of only producing identical matches in the alignment process [49]. In the mappings to the *Tc1-2_Xt* sequence, normalized frequency of reads was summed up only to the 5′-end nucleotide position (Figure 4B) or to all nucleotide positions (Figure 4D). Sequence logos were obtained from Weblogos server (http://weblogo.berkeley.edu/).

## Supporting Information

Figure S1 ***Tc*** **1-like elements nucleotide sequence alignment in the** ***X. tropicalis*** **genome.** All against all pairwise alignments of representative nucleotide sequences of *Tc*1-like elements of *X.tropicalis* were performed using ClustalW with default parameters and subsequently clustered by single linkage algorithm. Arrow indicates *Tc1-2_Xt*.
(TIF)

Figure S2 **Protein sequence comparison of** ***Tc*** **1-like elements in several species.** All against all pairwise alignments of representative available protein sequences of *Tc*1-like elements of several species were performed using ClustalW with default parameters and subsequently clustered by single linkage algorithm. Bracket indicates the cluster of *Tc1-2_Xt*.
(TIF)

Figure S3 **Regulated expression of the sense strand of** ***Tc1-2_Xt*** **during** ***X. tropicalis*** **development.** *In situ* hybridization with an antisense probe to specifically detect the sense strand of *Tc1-2_Xt* this element at stage 6 (**A**), 10 (**B**), 12 dorsal view and (**C**), ventral view (**D**), 18 (**E**) and 21 (**F**). (**G**) Transverse section of a stage 21 embryo and (**H**) close-up image of the neural tube.
(TIF)

Figure S4 **Regulated expression of** ***TXr*** **during** ***X. tropicalis*** **development.** *In situ* hybridization with a sense probe to specifically detect the antisense strand of *TXr* during *X. tropicalis* development. **(A)** st 6 animal view (maternal stages), **(B)** st 10 vegetal view with dorsal blastopore lip at the left, **(C)** st 13 dorsal view, **(D)** st 13 ventral view, **(E)** st 18 dorsal view and **(F)** st 25 dorsal view.
(TIF)

Figure S5 **Regulated expression of** ***TXr*** **during** ***X. laevis*** **development.** *In situ* hybridization with an antisense probe to specifically detect the sense strand of *TXr* during *X. laevis* development. **(A)** st 4 animal view, **(B)** st 10 lateral view with dorsal blastopore lip at the left, **(C)** st 11 lateral view with dorsal side at the top, **(D)** st 15 dorsal view, **(E)** st 22 dorsal view and **(F)** st 27 lateral view.
(TIF)

Figure S6 **Regulated expression of both strands of** ***TXz*** **during** ***X.laevis*** **development.** *In situ* hybridization with sense and antisense probes to specifically detect the antisense and sense strands, respectively, at different stages. **(A)** and **(E)** st 4 animal view, (**B**) st 15 lateral view, dorsal blastopore lip at the left, **(C)** st 22 dorsal view, **(D)** st 27 lateral view, **(F)** st 10 lateral view, **(G)** st 15 dorsal view, **(H)** st 20 dorsal view, **(I)** st 22 dorsal view and **(J)** st 28 lateral view.
(TIF)

Figure S7 **Analysis of small RNAs mapped to** ***Tc*** **1-like elements** ***in X. tropicalis*** **.** Weblogos of small RNAs mapped to *Eagle*, *Jumpy* and *Maya* according to the orientation of the transposon sequence (**A**, **C**, **E**). Histogram of the length of small RNAs mapped to *Eagle*, *Jumpy* and *Maya* (**B**, **D**, **F**).
(TIF)

Figure S8 **Expression of** ***Tc1-2_Xt*** **-derived piRNAs during** ***X. tropicalis*** **development**. **(A,B)** qPCR for piRNAs *p845* and *p910* at different stages of development. **(C)** qPCR for piRNAs *p845* and *p910* in dorsal (DMZ) and ventral (VMZ) explants of gastrula stage. Ratio values were obtained by normalization against a piRNA control.
(TIF)

Table S1 **Analysis of small RNAs mapped to** ***Tc*** **1-like elements in** ***X. tropicalis*** **.** Summary of the number of reads for each element. Only small RNA sequences mapped specifically to one *Tc*1-like element were considered for these analyses. The number of reads was normalized against the total number of reads obtained at the gastrula stage. Sequences mapped to inverted repeats were considered only once for each element. The percentage of reads in PIWI libraries is included for each element.
(DOC)

Table S2 Primer and LNA sequences used in this study.
(XLS)

File S1 Representative nucleotides sequences of *Tc1*-like elements of *X. tropicalis* used in this study.
(DOC)

File S2 Protein sequences of *Tc1*-like elements of several species used in this study.
(DOC)
