# Supplementary figures and images for: Expression of Transposable Elements in Neural Tissues during Xenopus Development
Source: PLoS One. 2011 Jul 26;6(7):e22569. doi: 10.1371/journal.pone.0022569 (PMC3144230; doi:10.1371/journal.pone.0022569)

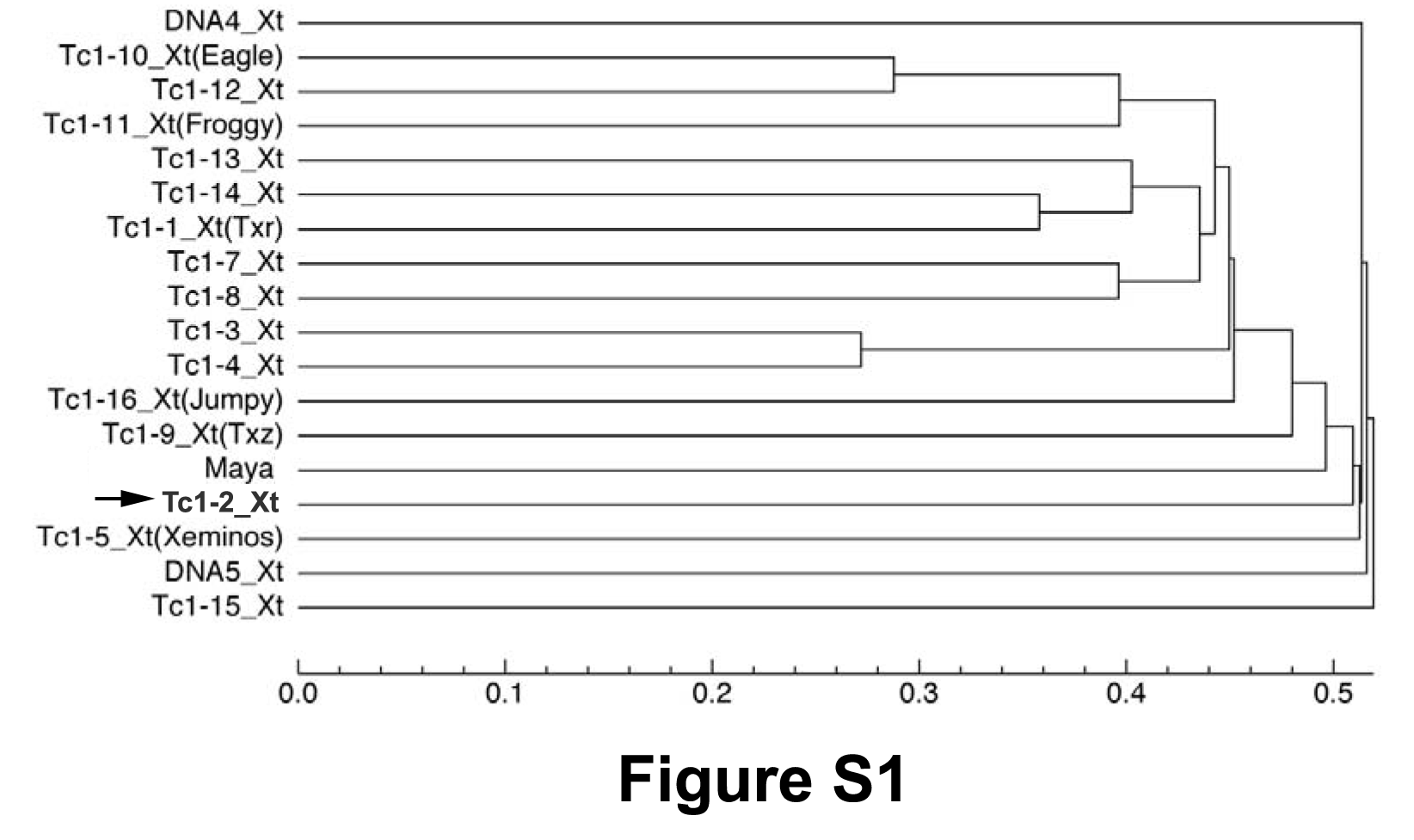

Supplement: Figure S1 — Tc 1-like elements nucleotide sequence alignment in the X. tropicalis genome. All against all pairwise alignments of representative nucleotide sequences of Tc1-like elements of X.tropicalis were performed using ClustalW with default parameters and subsequently clustered by single linkage algorithm. Arrow indicates Tc1-2_Xt. (TIF) [file pone.0022569.s001.tif]

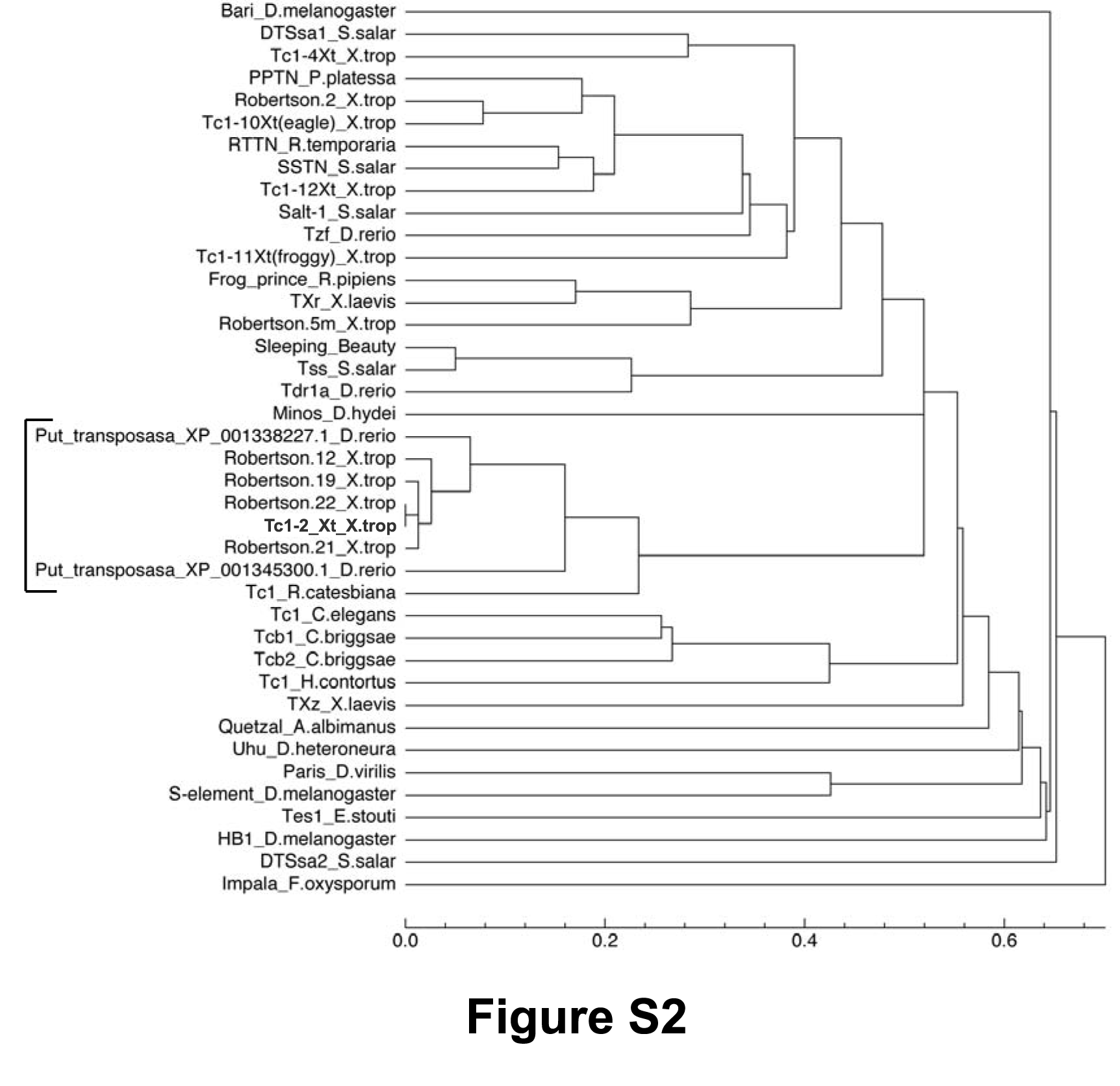

Supplement: Figure S2 — Protein sequence comparison of Tc 1-like elements in several species. All against all pairwise alignments of representative available protein sequences of Tc1-like elements of several species were performed using ClustalW with default parameters and subsequently clustered by single linkage algorithm. Bracket indicates the cluster of Tc1-2_Xt. (TIF) [file pone.0022569.s002.tif]

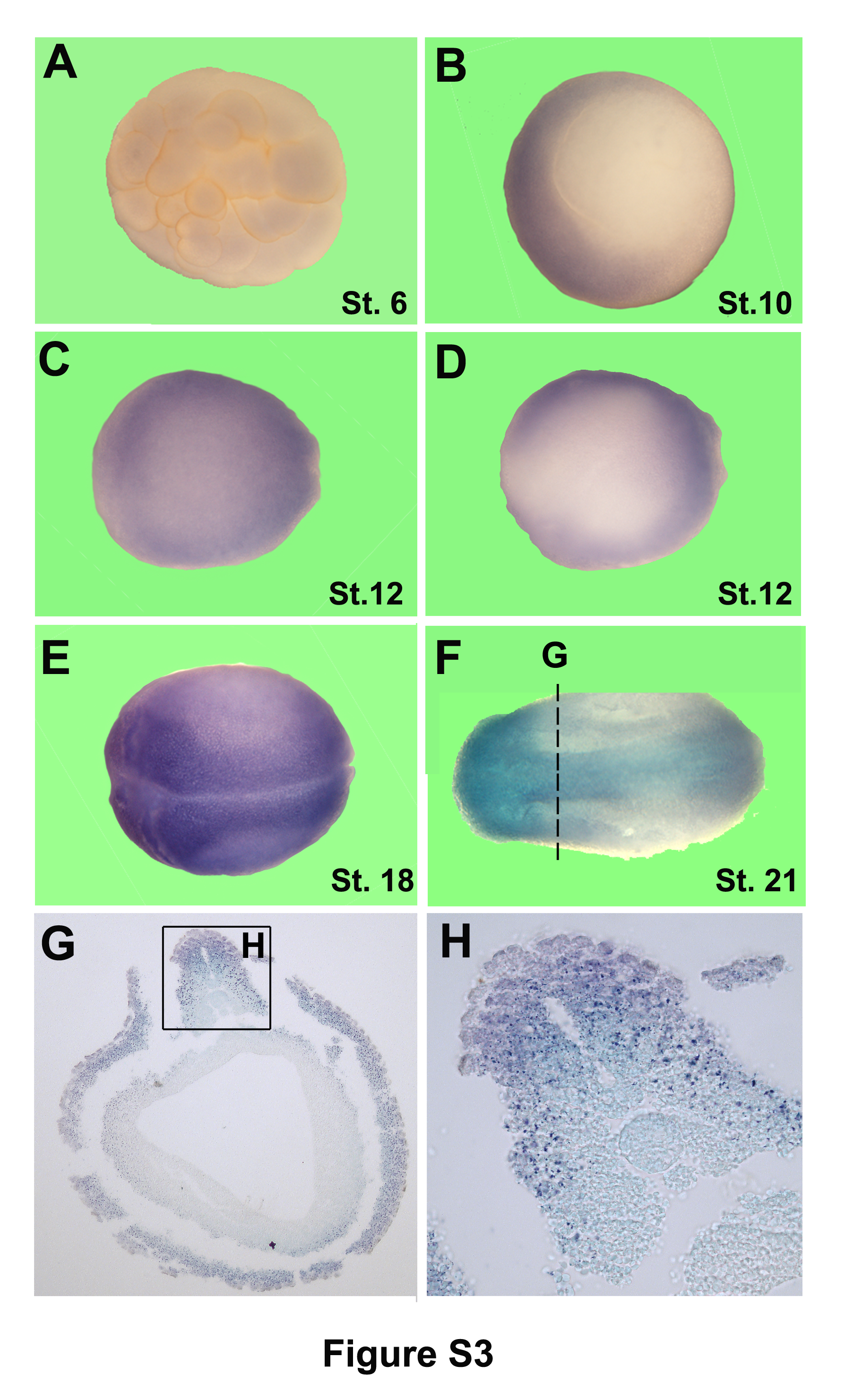

Supplement: Figure S3 — Regulated expression of the sense strand of Tc1-2_Xt during X. tropicalis development. In situ hybridization with an antisense probe to specifically detect the sense strand of Tc1-2_Xt this element at stage 6 (A), 10 (B), 12 dorsal view and (C), ventral view (D), 18 (E) and 21 (F). (G) Transverse section of a stage 21 embryo and (H) close-up image of the neural tube. (TIF) [file pone.0022569.s003.tif]

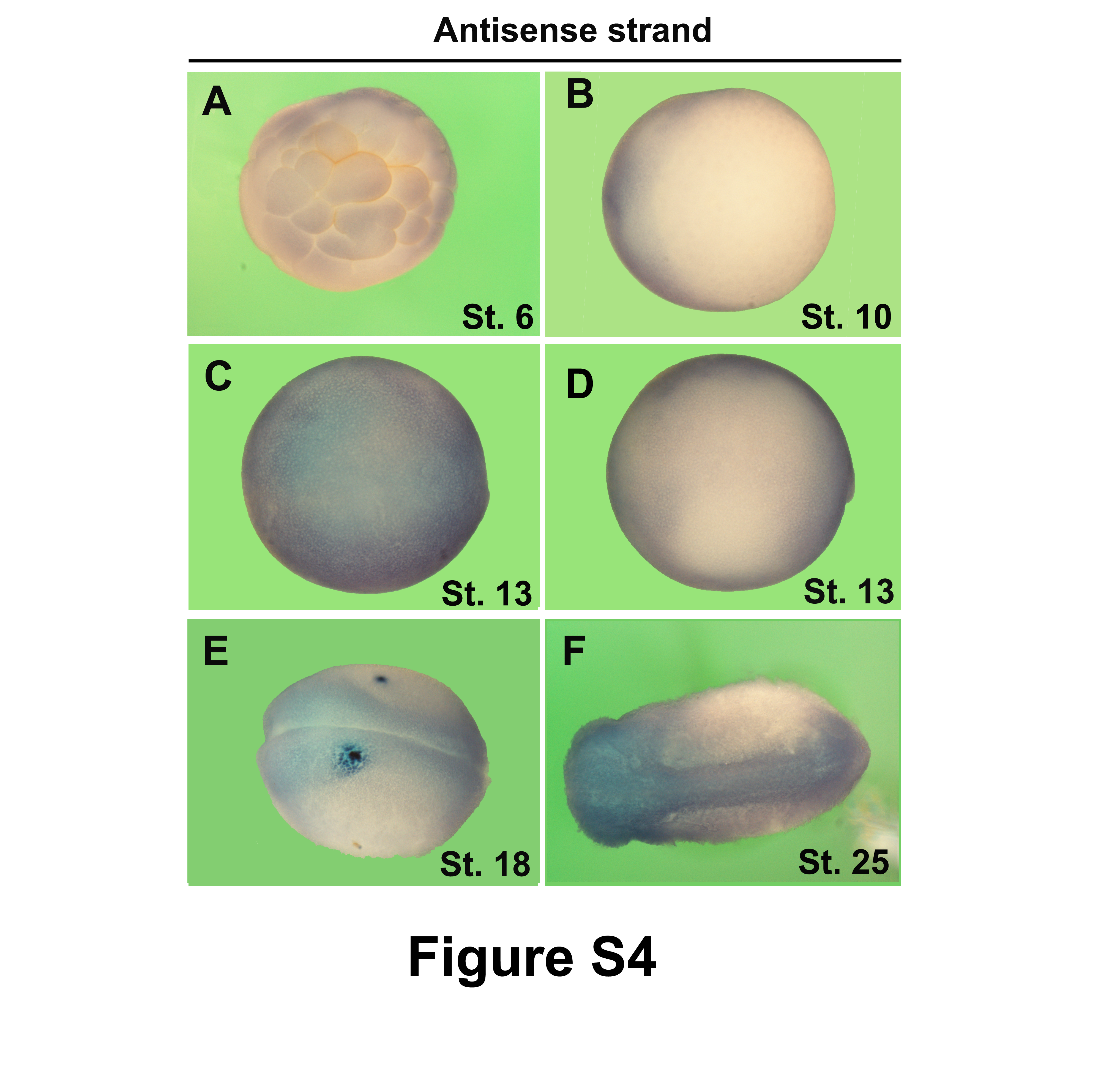

Supplement: Figure S4 — Regulated expression of TXr during X. tropicalis development. In situ hybridization with a sense probe to specifically detect the antisense strand of TXr during X. tropicalis development. (A) st 6 animal view (maternal stages), (B) st 10 vegetal view with dorsal blastopore lip at the left, (C) st 13 dorsal view, (D) st 13 ventral view, (E) st 18 dorsal view and (F) st 25 dorsal view. (TIF) [file pone.0022569.s004.tif]

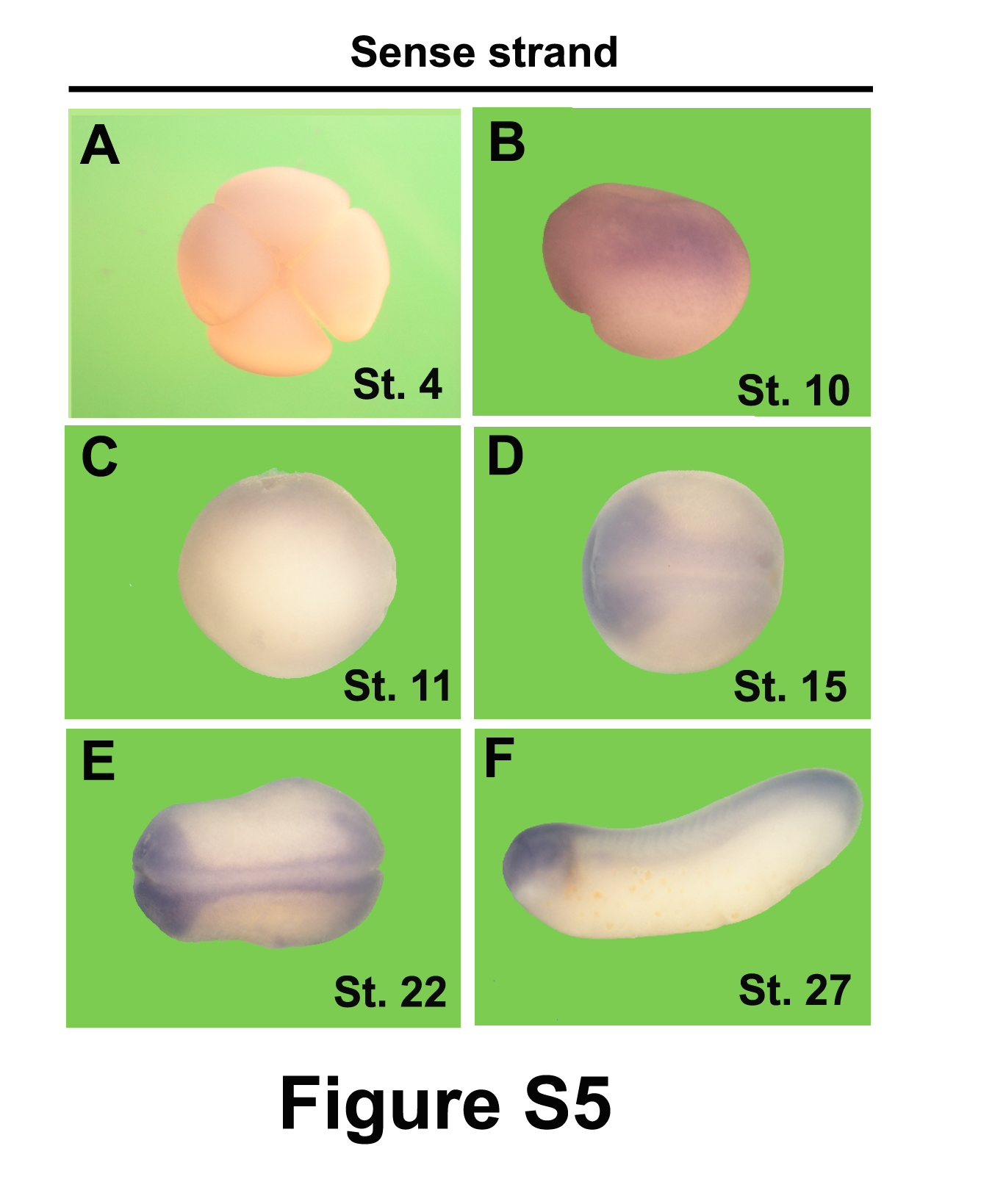

Supplement: Figure S5 — Regulated expression of TXr during X. laevis development. In situ hybridization with an antisense probe to specifically detect the sense strand of TXr during X. laevis development. (A) st 4 animal view, (B) st 10 lateral view with dorsal blastopore lip at the left, (C) st 11 lateral view with dorsal side at the top, (D) st 15 dorsal view, (E) st 22 dorsal view and (F) st 27 lateral view. (TIF) [file pone.0022569.s005.tif]

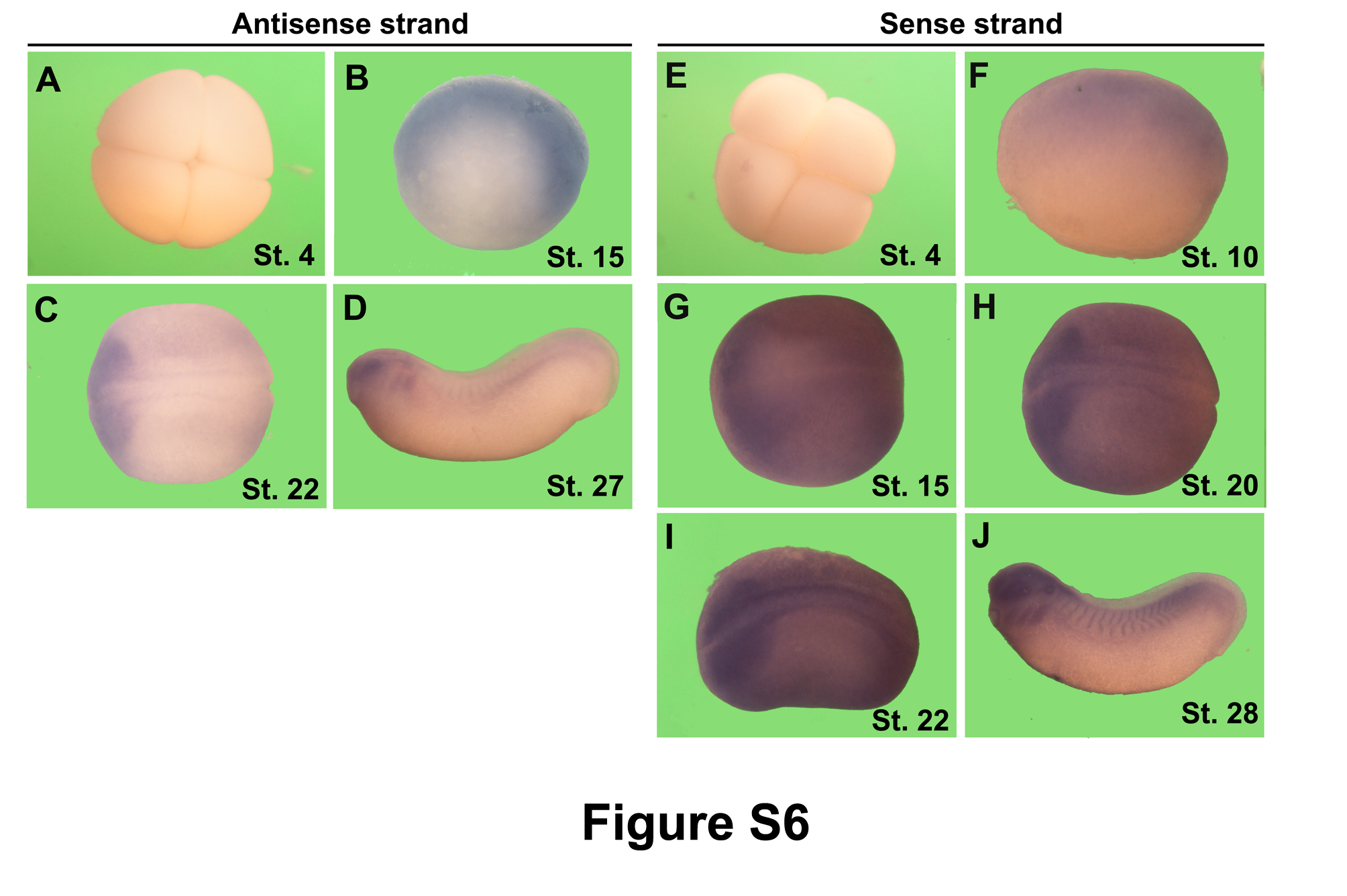

Supplement: Figure S6 — Regulated expression of both strands of TXz during X.laevis development. In situ hybridization with sense and antisense probes to specifically detect the antisense and sense strands, respectively, at different stages. (A) and (E) st 4 animal view, (B) st 15 lateral view, dorsal blastopore lip at the left, (C) st 22 dorsal view, (D) st 27 lateral view, (F) st 10 lateral view, (G) st 15 dorsal view, (H) st 20 dorsal view, (I) st 22 dorsal view and (J) st 28 lateral view. (TIF) [file pone.0022569.s006.tif]

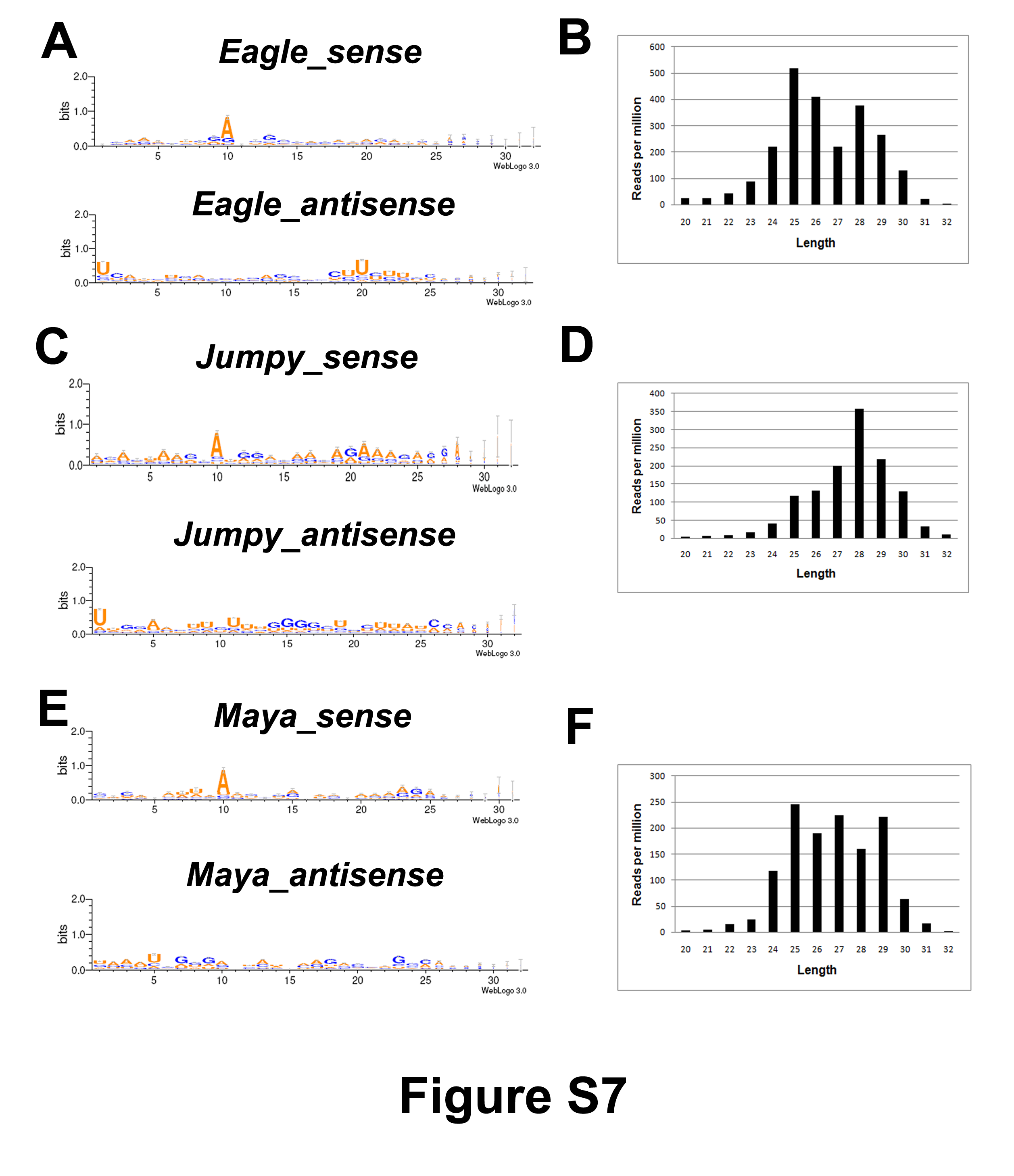

Supplement: Figure S7 — Analysis of small RNAs mapped to Tc 1-like elements in X. tropicalis . Weblogos of small RNAs mapped to Eagle, Jumpy and Maya according to the orientation of the transposon sequence (A, C, E). Histogram of the length of small RNAs mapped to Eagle, Jumpy and Maya (B, D, F). (TIF) [file pone.0022569.s007.tif]

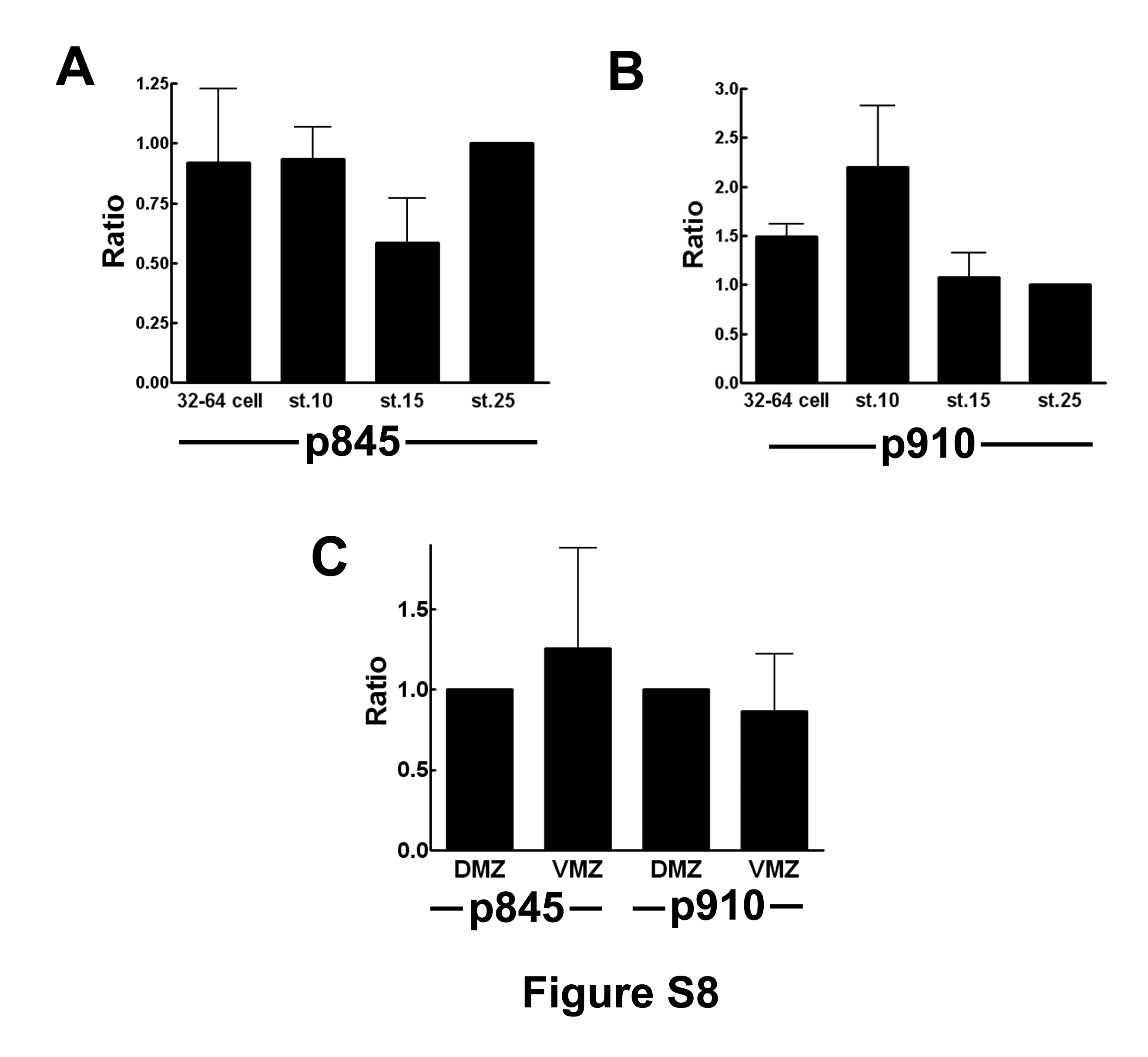

Supplement: Figure S8 — Expression of Tc1-2_Xt -derived piRNAs during X. tropicalis development. (A,B) qPCR for piRNAs p845 and p910 at different stages of development. (C) qPCR for piRNAs p845 and p910 in dorsal (DMZ) and ventral (VMZ) explants of gastrula stage. Ratio values were obtained by normalization against a piRNA control. (TIF) [file pone.0022569.s008.tif]
