# Supplementary material for: Expression of Transposable Elements in Neural Tissues during Xenopus Development
Source: PLoS One. 2011 Jul 26;6(7):e22569. doi: 10.1371/journal.pone.0022569 (PMC3144230; doi:10.1371/journal.pone.0022569)
Supplement: Table S1 — Analysis of small RNAs mapped to Tc 1-like elements in X. tropicalis . Summary of the number of reads for each element. Only small RNA sequences mapped specifically to one Tc1-like element were considered for these analyses. The number of reads was normalized against the total number of reads obtained at the gastrula stage. Sequences mapped to inverted repeats were considered only once for each element. The percentage of reads in PIWI libraries is included for each element. (DOC) [file pone.0022569.s009.doc]

| Tc1 | Total reads | Reads included in other Tc1 element | Final reads | Reads per million | Sense | Antisense | Inverted repeats | % sense | % antisense | % IR | % found in piwi libraries |
| --- | --- | --- | --- | --- | --- | --- | --- | --- | --- | --- | --- |
| Tc1-5-Xt(Xeminos) | 100714 | 0 | 100714 | 5737,67 | 137,75 | 5460,85 | 139,06 | 2,4 | 95,2 | 2,4 | 80,12 |
| Tc1-2-Xt | 49815 | 0 | 49815 | 2837,96 | 92,92 | 2675,93 | 69,10 | 3,3 | 94,3 | 2,4 | 80,89 |
| Tc1-10-Xt(Eagle) | 41263 | 16 | 41247 | 2349,84 | 89,16 | 2242,85 | 17,83 | 3,8 | 95,4 | 0,8 | 80,43 |
| Maya | 22617 | 0 | 22617 | 1288,49 | 69,67 | 1218,76 | 0,06 | 5,4 | 94,6 | 0,0 | 80,92 |
| Tc1-16-Xt(Jumpy) | 22311 | 0 | 22311 | 1271,06 | 97,36 | 1159,17 | 14,53 | 7,7 | 91,2 | 1,1 | 76,53 |
| Tc1-1-Xt(Txr) | 18788 | 0 | 18788 | 1070,35 | 54,75 | 1010,36 | 5,24 | 5,1 | 94,4 | 0,5 | 74,43 |
| Tc1-15-Xt | 15071 | 0 | 15071 | 858,59 | 49,51 | 809,09 | 0,00 | 5,8 | 94,2 | 0,0 | 68,79 |
| Tc1-3-Xt | 15022 | 121 | 14901 | 848,91 | 46,09 | 784,02 | 18,80 | 5,4 | 92,4 | 2,2 | 64,55 |
| Tc1-8-Xt | 13789 | 0 | 13789 | 785,56 | 72,64 | 712,92 | 0,00 | 9,2 | 90,8 | 0,0 | 63,77 |
| Tc1-11-Xt(Froggy) | 12441 | 0 | 12441 | 708,76 | 55,32 | 652,70 | 0,74 | 7,8 | 92,1 | 0,1 | 65,15 |
| Tc1-4-Xt | 10023 | 78 | 9945 | 566,57 | 74,40 | 476,78 | 15,38 | 13,1 | 84,2 | 2,7 | 58,19 |
| Tc1-9-Xt(Txz) | 7967 | 0 | 7967 | 453,88 | 128,52 | 320,74 | 4,61 | 28,3 | 70,7 | 1,0 | 52,89 |
| Tc1-12-Xt | 3454 | 16 | 3438 | 195,86 | 46,94 | 148,92 | 0,00 | 24,0 | 76,0 | 0,0 | 52,91 |
| Tc1-14-Xt | 2045 | 0 | 2045 | 116,50 | 55,94 | 53,78 | 6,78 | 48,0 | 46,2 | 5,8 | 48,31 |
| Tc1-13-Xt | 583 | 43 | 540 | 30,76 | 6,32 | 24,44 | 0,00 | 20,6 | 79,4 | 0,0 | 11,48 |
| DNA5-Xt | 94 | 0 | 94 | 5,36 | 1,25 | 4,10 | 0,00 | 23,4 | 76,6 | 0,0 | 8,51 |
| DNA4-Xt | 26 | 0 | 26 | 1,48 | 0,91 | 0,57 | 0,00 | 61,5 | 38,5 | 0,0 | 0 |
| Tc1-7-Xt | 1 | 0 | 1 | 0,06 | 0,00 | 0,06 | 0,00 | 0,0 | 100,0 | 0,0 | 0 |
| ef1a | 27 | 0 | 27 | 1,54 | 1,48 | 0,06 | 0,00 | 96,3 | 3,7 | 0,0 | 0 |
| odc | 5 | 0 | 5 | 0,28 | 0,28 | 0,00 | 0,00 | 100,0 | 0,0 | 0,0 | 0 |

**Table S1**
