# Supplementary material for: Expression of Transposable Elements in Neural Tissues during Xenopus Development
Source: PLoS One. 2011 Jul 26;6(7):e22569. doi: 10.1371/journal.pone.0022569 (PMC3144230; doi:10.1371/journal.pone.0022569)
Supplement: File S1 — Representative nucleotides sequences of Tc1-like elements of X. tropicalis used in this study. (DOC) [file pone.0022569.s011.doc]

**File S1**

>Tc1-1_Xt(Txr)

CAGTGGTGTGAAAAACTATTTGCCCCCTTCCTGATTTCTTATTCTTTTGCATGTTTGTCACACTTAAATGTTTCTGCTCATCAAAAACCGTTAACTATTAGTCAAAGATAACATAATTGAACACAAAATGCAGTTTTTAAATGAAGGTTTACGTTATTAAGGGAGAAAAAAAACTCCAAATCTACATGGCCCTGTGTGAAAAAGTGATTGCCCCCCTTGTTAAAAAATAACTTAACTGTGGTTTATCACATTTCAATTTTCAATTTCAATATCAATTTCTGTAGTCACCCCCAGGCCTGATTACTGCCACACCTGTTTCAATCAAGAAATCACTTAAATAGGAGCTACCTGACACAGAGAAGTAGACCAAAAGCACCTCAAAAGCTAGACATCATGCCAAGATCCAAAGAAATTCAGGAACAAATGAGAACAAAAGTAATTGAGATCTATCAGTCTGGTAAAGGTTATAAAGCCATTTCTAAAGCTTTGGGACTCCAGCGAACCACAGTGAGAGCCATTATCCACAAATGGCAAAAACATGGAACAGTGGTGAACCTTCCCAGGAGTGGCCGGCCGACCAAAATTACCCCAAGAGCGCAGAGACAACTCATCCGAGAGGCCACAAAAGACCCCAGGACAACATCTAAAGAACTGCAGGCCTCACTTGCCTCAATTAAGGTCAGTGTTCACGACTCCACCATAAGAAAGAGACTGGGCAAAAACGGCCTGCATGGCAGATTTCCAAGGCGCAAACCACTTTTAAGCAAAAAGAACATTANGGCTCGTCTCAATTTTGCTAAAAAACATCTCAATGATTGCCAAGACTTTTGGGAAAATACCTTGTGGACCGACGAGACAAAAGTTGAACTTTTTGGAAGGTGCGTGTCCCGTTACATCTGGCGTAAAAGTAACACAGCATTTCAGAAAAAGAACATCATACCAACAGTAAAATATGGTGGTGGTAGTGTGATGGTCTGGGGTTGTTTTGCTGCTTCAGGACCTGGAAGGCTTGCTGTGATAGATGGAACCATGAATTCTACTGTCTACCAAAAAATCCTGAAGGAGAATGTCCGGCCATCTGTTCGTCAACTCAAGCTGAAGCGATCTTGGGTGCTGCAGCAGGACAATGACCCAAAACACACCAGCAAATCCACCTCTGAATGGCTGAAGAAAAACAAAATGAAGACTTTGGAGTGGCCTAGTCAAAGTCCTGACCTGAATCCTATTGAGATGTTGTGGCATGACCTTAAAAAGGCGGTTCATGCTAGAAAACCCTCAAATAAAGCTGAATTACAACAATTCTGCAAAGATGAGTGGGCCAAAATTCCTCCAGAGCGCTGTAAAAGACTCGTTGCAAGTTATCGCAAACGCTTGATTGCAGTTATTGCTGCTAAGGGTGGCCCAACCAGTTATTAGGTTCAGGGGGCAATTACTTTTTCACACAGGGCCATGTAGGTTTGGATTTTTTTTCTCCCTAAATAATAAAAACCCTCATTTAAAAACTGCATTTTGTGTTTACTTGTGTTATCTTTGACTAATAGTTAAATGTGTTTGATGATCAGAAACATTTTGTGTGACAAACATGCAAAAGAATAAGAAATCAGGAAGGGGGCAAATAGTTTTTCACACCACTG

>Tc1-2_Xt(this work)

CAGGTCCTTTTCAAAAAATTAGCATATTGTGATAAAGTTCATTATTTTCTGTAATGTACT

GATAAACATTAGACTTTCATATATTTTAGATTCATTACACACAACTGAATTATTTCAAGC

CTTTTATTGTTTTAATATTGATGATTGTGGCATACAGCTCATGAAAACCCACAATTCCTA

TCTCAAAAAATTAGCATATCATGAAAAGGTTCTCTAAACGAGCTATTAACCTAATCATCT

GAATCAACTAATTAACTCTAAACACCTGCAAAAGATTCCTGAGGCTTTTAAAAACTCCCA

GCCTGGTTCATTACTAAAAACCGCAATCATGGGTAAGACTGCCGACCTGACTGCTGTCCA

GAAGGCCATCATTGACACCCTCAAGCAAGAGGGTAAGACACAGAAAGAAATTTCTGAACG

AATAGGCTGTTCCCAGAGTGCTGTATCAAGGCACCTCAGTGGGAAGTCTGTGGGAAGGAA

AAAGTGTGGCAGAAAACGCTGCACAACGAGGAGAGGTGACCGGGCCCTGAGGAAGATTGT

GGAGAAGGACCGATTCCAGACCTTGGGGGACCTGCGGAAGCAGTGGACGGAGTCTGGAGT

AGAAACATCCAGAGCCACCATGTACAGGCGTGTGCAGGAAATGGGCTACAGGTGCCGCAT

TCCCCAGGTCAAGCCACTTTTGAACCAGAAACAGCGGCAGAAGCGCCTGACCTGGGCTAC

AGAGAAGCAGCACTGGACTGTTGCTCAGTGGTCCAAATTACTTTTTTCGGATGAAAGCAA

ATTTTGCATGTCATTCAGAAATCAAGGTGCCAGAGTCTGGAGGAAGACTGGGGAGAGAGA

AATGCCAAAATGCCTGAAGTCCAGTGTCAAGTACCCACAGTCAGTGATGGTCTGGGGTGC

CATGTCAGCTGCTGGTGTTGGTCCACTGTGTTTTATCAAGGGCAGGGTCAATGCAGCTAG

CTATCAGGAGATTTTGGAGCACTTCATGCTTCCATCTGCTGAAAAGCTTTATGGAGATGA

AGATTTCATTTTTCAGCACGACCTGGCACCTGCTCACAGTGCCAAAACCACTGGTAAATG

GTTTACTGACCATGGTATTACTGTGCTCAACTGGCCTGCCAACTCTCCTGACCTGAACCC

CATAGAGAATCTGTGGGATATTGTGAAGAGAAAGTTGAGAGACACAAGACCCAACAGTCT

GGATGAGCTTAAGGCCGCTATTGAAGCATCCTGGGCCTCCATAACACCTCAGCAGTGCCA

CAGGCTGATTGCCTCCATGCCACGCCGCATTGAAGCAGTAATTTCTGCAAAAGGATTCCC

GACCAAGTATTGAGTGCATAACTGAACATAATTATTTGAAGGTTGATTTTTTTTGTATTA

AAAACACTTTTCTTTTATTGGTCGGATGAAATATGCTAATTTTTTGAGATAGGAATTTTG

GGTTTTCATGAGCTGTATGCCACAATCATCAATATTAAAACAACAAAAGGCTTGAACTAC

TTCAGTTGTGTGTAATGAATCTAATGAAAATAATGAACTTTATCACAATATGCTAATTTT

TTGAAAAGGACCTG

>Tc1-3_Xt

CAGTGGAGGAAATAATTATTTGACCCCTCACTGATTTTGTAAGTTTGTCCAATGACAAAGAAATGAAAAGTCTCAGAACAGTATCATTTCAATGGTAGGTTTATTTTAACAGTGGCAGATAGCACATCAAAAGGAAAATCGAAAAAATAACTTTAAATAAAAGATAGCAACTGATTTGCATTTCATTGAGTGAAATAAGTTTTTGAACCCCTACCAACCATTAAGAGTTCTGGCTCCCACAGAGTGGTTAGACACTTCTACTCAATTAGTCAACCTCATTAAGGACACCTGTCTTAACTAGTCACCTGTATAAAAGACACCTGTCCACAGAATCAATCAATCAAGCAGACTCCAAACTCTCCAACATGGGAAAGACCAAAGAGCTGTCCAAGGATGTCAGAGACAAAATTGTAGACCTGCACAAGGCTGGAATGGGCTACAAAACCATTAGCAAGAAGCTGGGAGAGAAGGTGACAACTGTTGGTGCGATTGTTCGAAAATGGAAGGAGCACAAAATGACCATCAATCGACCTCGCTCTGGGGCTCCACGCAAGATCTCACCTCGTGGGGTGTCAATGATTCTGAGAAAGGTGAAAAAGCATCCTAGAACTACACGGGAGGAGTTAGTTAATGACCTCAAATTAGCAGGGACCACAGTCACCAAGAAAACCATTGGAAACACATTACACCGCAATGGATTAAAATCCTGCAGGGCTCGCAAGGTCCCCCTGCTCAAGAAGGCACATGTGCAGGCCCGTCTGAAGTTTGCCAATGAACACCTGAATGATTCTGTGAGTGACTGGGAGAAGGTGCTGTGGTCTGATGAGACCAAAATAGAGCTCTTTGGCATTAACTCAACTCGCTGTGTTTGGAGGAAGAAAAATGCTGCCTATGACCCCCAAAACACCGTCCCCACCGTCAAGCATGGGGGTGGAAACATTTTGCTTTGGGGGTGTTTTTCTGCTAAGGGCACAGGACAACTTATTCGCATTAACGGGAAAATGGACGGAGCCATGTATCGTGAAATCCTGAACGACAACCTCCTTCCCTCTGCCAGGAAACTGAAAATGGGTCGTGGATGGGTGTTCCAGCACGACAATGACCCAAAACATACAGCAAAGGCAACAAAGGAGTGGCTCAAGAAGAAGCACATTAAGGTCATGGAGTGGCCTAGTCAGTCTCCGGACCTTAATCCAATAGAAAACCTATGGAGGGAGCTCAAGCTCAGAGTTGCACAGAGACAGCCTCGAAACCTTAGGGATTTAGAGATGATCTGCAAAGAGGAGTGGACCAACATTCCTCCTAAAATGTGCGCAAACTTGGTCATCAATTACAAGAAACGTTTGACCTCTGTGCTTGCAAACAAGGGTTTTTCCACTAAGTATTAAGTCTTTTTTTGTTAGAGGGTTCAAAAACTTATTTCACTCAATGAAATGCAAATCAGTTGCTATCTTTTATTTAAAGTTATTTTTTCGATTTTCCTTTTGATGTGCTATCTGCCACTGTTAAAATAAACCTACCATTGAAATGATACTGTTCTGAGACTTTTCATTTCTTTGTCATTGGACAAACTTACAAAATCAGTGAGGGGTCAAATAATTATTTCCTCCACTG

>Tc1-4_Xt

CAGTGAGGAACATAAGTATTTGAACACCCTGCGATTTTGCAAGTTCTCCCACTTAGAAATCATGGAGGGGTCTGAAATTCACATTGTAGGTGCATTCCCACTGTGAGAGACAGCATTTAAAAAAAAAATTCAGGAAATCACATTGTATGATTTTTAAAGAATGTATTTGTATTGCACTGCTGCACATAAGTATTTGAACACCTGGCAATCAGCAAGAATTCTGGCTCTCAAAGACCTGTTACTCTGCCTTTAAANAGTCCACCTCTACTCCACTCATTAATCTAAATTAGTAGCACCTGTCTGAGCTCTTTAAAGACACCTGTCCACCCCACAGTCAGTCAGACTCCAACTACTACCATGGGCAAGACCAAAGAGCTGTCAAAAGACACCAGAGACAAAATTGTGGACCTCCACAAGGCTGGAAAGGGCTACGGGGCAATTGCCAAGCAGCTTGGTGAAAATAGATCAACTGTTGGAGCAATTGTTAGAAAATGGAAGAGGCTAAAGACGACTGTCAGTCTCCCTAGGACTGGGGCTCCATGCAAGATCTCACCTCGTGGGGTATCACTGATGATAAGAAAGGTGAGGAATCAGCCCAGAACTACAAGGGAGGAGCTGGTCAATGACATGAAGAGAGCTGGGACCACAGTTTCAAAGGTCACTGTCGGTAGAACACTACGCCGTCATGGTTTCAAATCATGCATTGCACGGAAGGTTCCCCTGCTCAAGTCATCACATGTCCAGGCCCGTCTGAAGTTTGCCAATGACCATCTGGATGATCCAGAGGAGGCATGGGAGAAAGTCATGTGGTCAGATGAGACCAAAGTAGAACTTTTTGGTCTAAACTTCACTCGCCGTGTTTGGAGGAAAAATAAGGATGAGTTGCATCCCAAGAACACCATCCCTACTGTGAAGCATGGGGGTGGTAACATCATGCTTTGGGGGTGCTTTTCTGCGAAGGGGACAGGACGACTGCACTGTATTAAGGAGAGGATGAATGGGGCCATGTATTGTGAGATTTTGAGCAACAACCTCCTTCCCTCAGTCAGAGCATTGAAGATGGGTCGTGGCTGGGTCTTCCAACATGACAATGACCCGAAGCACACAGCCAGGATAACCAAGGAGTGGCTCCGTAAGAGGCATATCAAGGTTCTGGAGTGGCCTAGCCAGTCTCCAGACCTAAATCCAATAGAAAATCTTTGGAGGGAGCTGAAACTCCGTGTTGCTCAGCGACAGCCCCGAAACCTGACAGATCTAGAGGAGATCTGTGTGGAGGAGTGGGCCAAAATCCCTGTTGCAGTGTGTGCAAACCTGGTCAAGAACTACAGGAAACGTTTGACCTCTGTAATTGCAAACAAAGGCTTCTGTACCAAATATTAACACTGATTTTCTCAGGTGTTCAAATACTTATGTGCAGCAGTGCAATACAAATACATTCTTTAAAAATCATACAATGTGATTTCCTGAATTTTTTTTTTAAATGCTGTCTCTCACAGTGGGAATGCACCTACAATGTGAATTTCAGACCCCTCCATGATTTCTAAGTGGGAGAACTTGCAAAATCGCAGGGTGTTCAAATACTTATGTTCCTCACTG

>Tc1-5_Xt(Xeminos)

CAAACCGGATTCCAAAAAAGTTGGGACACTAAACAAATTGTGAATAAAAACTGAACGCAATGATGTGGAGGTGCCAACTTCTAATATTTTATTCAGAATAGAACATAAATCACGGAACAAAAGTTTAAACTGAGAAAATGTACCATTTTAAGGGAAAAATATGTTGATTCAGAATTTCATGGTGTCAACAAATCCCAAAAAAGTTGGGACAAGTAGCAATAAGAGGCTGGAAAAAGTAAATTTGAGCATAACGAAGAGCTGGAAGACCAAATAACACTAATTAGGTCAATTGGCAACATGATTGGGTATAAAAAGAGCTTCTCAGAGTGGCAGTGTCTCTCAGAAGCCAAGATGGGTAGAGGATCACCAATTCCCACAATGTTGCGCAGAAAGATAGTGGAGCAATATCAGAAAGGTGTTACCCAGCGAAAAATTGCAAAGATTTTGCATCTATCATCATCAACTGTGCATAACATCATCCGAAGATTCAGAGAATCTGGAACAATCTCTGTGCGTAAGGGTCAAGGCCGTAAAACCATACTGGATGCCCGTGATCTCCGGGCCCTTAAACGACACTGCACCACAAACAGGAATGCTACTGTAAAGGAAATCACAGAATGGGCTCAGGAATACTTCCAGAAACCATTGTCAGTGAACACAATCCACCGTGCCATCCGCCGTTGCCAGCTGAAACTCTACAGTGCAAAGAAGAAGCCATTTCTAAGCAAGATCCACAAGCTCAGGCGTTTTCACTGGGCCAGGGATCATTTAAAATGGAGTGTGGCAAAATGGAAGACTGTTCTGTGGTCAGACGAGTCACGATTCGAAGTTCTTTTTGGAAATCTGGGACGCCATGTCATCCGGACCAAAGAGGACAAGGACAACCCAAGTTGTTATCAACGCTCAGTTCAGAAGCCTGCATCTCTGATGGTATGGGGTTGCATGAGTGCGTGTGGCATGGGCAGCTTGCATGTCTGGAAAGGCAGCATCAATGCAGAAAAATATATTCAGGTTCTAGAACAACATATGCTCCCATCCAGACGTCATCTCTTTCAGGGAAGACCCTGCATTTTTCAACAAGATAATGCCAGACCACATTCTGCATCAATCACAACATCATGGCTGCGTAGGAGAAGGATCCGGGTACTGAAATGGCCAGTCTGCAGTCCAGATCTTTCACCTATAGAGAACATTTGGCGCATCATAAAGAGGAAGGTGCGACAAAGAAGGCCCAAGACGATTGAACAGTTAGAGGCCTGTATTAGACAAGAATGGGAGAGCATTCCTATTCCTAAACTTGAGAAACTGGTCTCCTCGGTCCCCAGACGTCTGTTGAGTGTTGTAAGAAGAAGGGGAGATGCCACACAGTGGTGAAAATGGCCTTGTCCCAACTTTTTTGGGATTTGTTGACACCATGAAATTCTGAATCAACATATTTTTCCCTTAAAATGGTACATTTTCTCAGTTTAAACTTTTGTTCCGTGATTTATGTTCTATTCTGAATAAAATATTAGAAGTTGGCACCTCCACATCATTGCGTTCAGTTTTTATTCACAATTTGTTTAGTGTCCCAACTTTTTTGGAATCCGGGTTG

>Tc1-7_Xt

CAGTTGAATGCAAAAGTTTGGGCACCCCTTGCCAAATNACATATTTAGTTAATTTTGTAAGTGAAAAGTAGTAAACAACTACTGCAGGAATCAGTGTGTTAAAAACAACATATTTGCAAATGTTAATGCACAGTTACATTTTATTTTACNAACTTTAAAACATAGGAACAAAGAAAATAGTAACTGTGGCATGTGCAAAAGTTTGGACACCCTTCCACTTGTCCAGTGATAAACACTGTTTTTGCAAGGTCCCTGACCCTAATTACCTCATTAGGCCTTAATAGCCATTAGGAGTTGTCACCTGTTGACAATTGCAGAGCTTAATAAAATCTCTGACACCCCAAACTTTGGGCTGTCACTCAACAACCATGGGCTCCTCTAAGCAACTGAGTGAGGATCTGAAAATGAAGCTAATTGATGCCTACAAAGCAGGGGAAGGCTATAAAAAGATTGCAAAACGCTTCCAGCTCACAATTTCCACTGTCCGTAATGTCATCAAGAAATGGCAGTTAAGGGGAACTGTGGAAGTCAAGGCAAGATCTGGAAGANCAAGAAAACTTTCAGAGAGAACTGCTCGTATGCTGGCCAGAAAGGCAAAGGCAAACCCTCATATGACTGCAAAGGACCTGCAGGAAGGTTTGGCTGACACAGGAGTGGTGTTGCACTGTTCCACGGTGCAGCGTTGCTTGCACAAACATGATCTGCATGGAAGAGTCATCAGGAGGAAGCCTTACCTGCAACCTCATCACAAACGTCAACGTCTGAGGTATGCAAAACAGCATCTAGACAAGCCAGAGGCCTTTTGGAAACAAGTGCTGTGGACTGATGAAGTAAAAATTNAACTCTTTGGCCACAATCACCAAAGGTTTGTTTGGAGAAAAAAAGGAGCAGCATTTGATGAAAAAAACACCTTGCCAACTGTTAAACATGGGGGTGGATCCATTATGCTTTGGGGTTGTGTGGCAGCCAGTGGCACAGGAAACATTGTACGTGTAGAGGGAAGAATGGATTCCACTAAATATCAGCAAATTCTGGATGCCAATGTGAAACAGTCAGCCAAGAAGCTGAAGCTGAAAAGGGGATGGCTCCTACAACAAGACAATGATCCTAAACATACCTCAAAAGCCACCATGAGCTACTTGAAGAAAAGCAAGCTGAAGGTTTTGGAATGGCCCTCACAGTCCCCTGACTTAAACATCATTGAAAATCTGTGGGTAGATCTTAAACATGCAAGATGGCCCAAGAAGATCTCGGAATTAGAAGTGATCTGCAAGGAAGAGTGGGCAAAAATCCCTACAACAAGAACTGAAAGACTCTTAGCTGGATACAAAAGGCATTTACAAGCTGTGATCTGTGCCAAAGGGGGTGTTACTAAATACTGACTTACTAGGGTGTCCAAACTTTTGCACATGCCACAATTACTATTTTCTTTGTTCCTGTGTTTTAAAGTTNGTGAAATAAAATGTAACTGTGCATTAACATTTGCAAATATGTTGTTTTTAACACACTGATTCCTGCAGTAGCTGTTTCTACTTTTCACTTACAAAATTAACTAAATATGTAATTTGGCAAGGGGTGCCCAAACTTTTGCATTCAACTG

>Tc1-8_Xt

CAGTCATATGAAAAAGTTTGGGAACCCCTCTCAGCCTGCATAATAATTTACTCCACTTTCAACAAAAAAAGATAACAGTGGTATGTCTTTCATTTCCCAGGAACATCTGAGTACTGGGGTGTTTTCTGAACAAAGATTTTTAGTGAAGCAGTATTTAGTTGTATGAAATTAAATCAAATGTGAAAAACTGGCTGTGCAAAAATTTGGGTACCCTTGTAATTTTGCTAATTTGAATGCATGTAACTGCTCAATACTGATTACTGGCAACACCAAATTGGTTGGATTAGCTTGTTAAGCCTTGAACTTCATAGGCAGGTGTGTCCAATCATGAGAAAAGGTATTTAAGGTGGCCAATTGCAAGTTGTGCTTCTGTTTGACTCTCCTCTGAAGAGTGACAGCATGGGATCCTCAAAGCAACTCTCAAAAGATCTGAAAACAAAGATTGTTCAGTATCATGGTTTAGGGGAAGGCTACAAAAAGCTATCTCAGAGGTTTAAACTGTCAGTTTCAACTGTAAGGAATGTAATCAGGAAATGGAAGGCCACAGGCACAGTTGCTGTAAAACCCAGGTCTGGCAGGCCAAGAAAAATACAGGAGCGGCATATGCGGAGGATTGTGAGAATGGTTACAGACAACCCAAAGATCACCTCCAAAGACCTGCAAGAACATCTTGCTGCAGATGGTGTATCTGTACATCGTTCTACAATTCAGCGCAATTTGCACAAAGAACATCTGTATGGCAGGGTGATGAGAAAGAAGCCCTTTCTGCACTCACGCCACAAACAGAGTCGCTTGTTGTATGCAAAAGCTCATTTAGACAAGCCACAGTCATTTTGGAACAAAGTGCTTTGGACTGATGAGACAAAAATTGAGTTATTTGGTCATAACAAAAAGCGCTTTGCATGGCGGAAGAAGAACACCGCATTCCAAGAAAAACACCTGCTACCTACTGTCAAATTTGGTGGAGGTTCCATCATGCTGTGGGGCTGTGTGGCTAGTTCAGGGACTGGGGCCCTTGTTAAAGTCGAGGGTCGGATGAATTCAACCCAATATCAACAAATTCTTCAGGATAATGTTCAAGCATCAGTCACAAAGTTGAAGTTACGCAGGGGTTGGATATTCCAACAAGACAATGACCCTAAACACAGTTGGAAATCTACAAAGGCATTTATGCAGAGGGAGAAGTACAATATTCTGGAATGGCCGTCACAGTCCCCCGACTTGAATATCATCGAAAATCTATGGGATGATTTGAAGCAGGCTGTCCATGCTCGGCAGCCATCAAATTTAACTGAACTGGAGAGATTTTGTATGGACGAATGGTCAAAAATACCGCCATCCAGAATCCAGACACTCATCAAAGGCTATAGGAGGCGTCTAGAGGCTGTTACATTTGCAAAAGGAGGCTCAACTAAGTATTGATGTAATATCTCTGTTGGGGTGCCCAAATTTATGCACCTGTCTAATTTTGTTATGATGCATATTGCATATTTTCTGTTAATCCAATAAACTTTATGTCACTGCTGAAATACTACTGTTTCCATAAGGCATGTTATATATTAAAAGGAAGTTGCTACTTTGAAAGCTCAGCCAATGATAAACAAAACTCCAAAGAATTAAGAGGGGTTCCCAAACTTTTTCATATGACTG

>Tc1-9_Xt(Txz)

CAGTTAGGTCCATAAATATTTGGACAGAGACAACTTTTTTCTAATTTTGGTTCTGTACATTACCACAATGAATTTTAAATGAAACAACTCAGATGCAGTTGAAGTGCAGACTTTCAGCTTTAATTCAGTGGGGTGAACAAAACGATTGCATAAAAATGTGAGGCCACTAAAGCATTTTTTTTAACACAATCCCTTCATTTCAAGGGCTCAAAAGTAATTGGACAATTGACTCAAAGGCTATTTCATGGGCAGGTGTTGGCAAGTCCGTCGTTATGTCATTATCAATTAAGCAGATAAAAGGCCTGGAGTTGATTTGAGGTGTGGTGCTTGCATGTGGAAGATTTTGCTGTGAACAGACAACATGCGGTCAAAGGAGCTCTCCATGCAGGTGAAAGAAGCCATCCTTAAGCTGCGAAAACAGAAAAAACCCATCCGAGAAATTGCTACAATATTAGGAGTGGCAAAATCTACAGTTTGGTACATCCTGAGAAAGAAAGCAAGCACTGGTGAACTCAGCAACGCAAAAAGACCTGGACGTCCACGGAAAACAACAGTGGTGGATGATCGTAGAATCATTTCCATGGTGAAGAGAAACCCCTTCACAACAGCCAACCAAGTGAACAACACTCTCCAGGGGGTAGGCGTATCGATATCCAAGTCTACCATAAAGAGAAGACTGCATGAAAGTAAATACAGAGGGTGCACTGCAAGGTGCAAGCCTCTCATAAGCCTCAAGAATAGAAAGGCTAGATTGGACTTTGCTAAAGAACATCTAAAAAAGCCAGCACAGTTCTGGAAAAACATTCTTTGGACAGATGAAACCAAGATCAACCTATACCAGAATGATGGCAAGAAAAAAGTATGGAGAAGGCGTGGAACAGCTCATTATCCAAAGCATACCACATCATCTGTAAAACACGGTGGAGGCAGTGTGATGGCTTGGGCGTGCATGGCTGCCAGTGGCACTGGGACACTAGTGTTTATTGATGATGTGACACAGGACAGAAGCAGCCGAATGAATTCTGAGGTGTTCAGAGACATACTGTCTGCTCAAATCCAGCTAAATGCAGTCAAATTGATTGGGCGGCGTTTCATGATACAGATGGACAATGACCCAAAACACACAGCCAAAGCAACCCAGGAGTTTATTAAAGCAAAGAAGTGGAAAATTCTTGAATGGCCAAGTCAGTCACCTGATCTTAACCCAATTGAGCATGCATTTCACTTGTTGAAGACTAAACTTCGGACAGAAAGGCCCACAAACAAACAGCAACTGAAAGCCGCTGCAGTAAAGGCCTGGCAGAGCATTAAAAAGGAGGAAACCCAGCATCTGGTGATGTCCATGAGTTCAAGACTTCAGGCTGTCATTGCCAGCAAAGGGTTTTCAACCAAGTATTAGAAATGAACATTTTATTTCCAGTTATTTAATTTGTCCAATTACTTTTGAGCCCCTGAAATGAAGGGATTGTGTTAAAAAAAATGCTTTAGTGGCCTCACATTTTTATGCAATCGTTTTGTTCACCCCACTGAATTAAAGCTGAAAGTCTGCACTTCAACTGCATCTGAGTTGTTTCATTTAAAATTCATTGTGGTAATGTACAGAACCAAAATTAGAAAAAAGTTGTCTCTGTCCAAATATTTATGGACCTAACTG

>Tc1-10_Xt(Eagle)

CAGTGGCTTGCAAAAGTATTCGGCCCCCTTGAACTTTTCCACATTTTGTCACATTACAGCCACAAACATGAATCAATTTTATTGGAATTCCACGTGAAAGACCAATACAAAGTGGTGTACACGTGAGAAGTGGAACGAAAATCATACATGATTCCAAACATTTTTTACAAATAAATAACTGCAAAGTGGGGTGTGCGTAATTATTCAGCCCCCTTTGGTCTGAGTGCAGTCAGTTGCCCATAGACATTGCCTGATGAGTGCTAATGACTAAATAGAGTGCACCTGTGTGTAATCTAATGTCAGTACAAATACAGCTGCTCTGTGACGGCCTCAGAGGTTGTCTAAGAGAATATTGGGAGCAACAACACCATGAAGTCCAAAGAACACACCAGACAGGTCAGGGATAAAGTTATTGAGAAATTTAAAGCAGGCTTAGGCTACAAAAAGATTTCCAAAGCCTTGAACATCCCACGGAGCACTGTTCAAGCGATCATTCAGAAATGGAAGGAGTATGGCACAACTGTAAACCTACCAAGACAAGGCCGTCCACCTAAACTCACAGGCCGAACAAGGAGAGCGCTGATCAGAAATGCAGCCAAGAGGCCCATGGTGACTCTGGACGAGCTGCAGAGATCTACAGCTCAGGTGGGGGAATCTGTCCATAGGACAACTATTAGTCGTGCACTGCACAAAGTTGGCCTTTATGGAAGAGTGGCAAGAAGAAAGCCATTGTTAACAGAAAACCATAAGAAGTCCCGTTTGCAGTTTGCCACAAGCCATGTGGGGGACACAGCAAACATGTGGAAGAAGGTGCTCTGGTCAGATGAGACCAAAATGGAACTTTTTGGCCAAAATGCAAAACGCTATGTGTGGCGGAAAACTAACACTGCACATCACTCTGAACACACCATCCCCACTGTCAAATATGGTGGTGGCAGCATCATGCTCTGGGGGTGCTTCTCTTCAGCAGGGACAGGGAAGCTGGTCAGAGTTGATGGGAAGATGGATGGAGCCAAATACAGGGCAATCTTGGAAGAAAACCTCTTGGAGTCTGCAAAAGACTTGAGACTGGGGCGGAGGTTCACCTTCCAGCAGGACAACGACCCTAAACATAAAGCCAGGGCAACAATGGAATGGTTTAAAACAAAACATATCCATGTGTTAGAATGGCCCAGTCAAAGTCCAGATCTAAATCCAATCGAGAATCTGTGGCAAGATCTGAAAACTGCTGTTCACAAACGCTGTCCATCTAATCTGACTGAGCTGGAGCTGTTTTGCAAAGAAGAATGGGCAAGGATTTCAGTCTCTAGATGTGCAAAGCTGGTAGAGACATACCCTAAAAGACTGGCAGCTGTAATTGCAGCAAAAGGTGGTTCTACAAAGTATTGACTCAGGGGGCTGAATAATTACGCACACCCCACTTTGCAGTTATTTATTTGTAAAAAATGTTTGGAATCATGTATGATTTTCGTTCCACTTCTCACGTGTACACCACTTTGTATTGGTCTTTCACGTGGAATTCCAATAAAATTGATTCATGTTTGTGGCTGTAATGTGACAAAATGTGGAAAAGTTCAAGGGGGCCGAATACTTTTGCAAGCCACTG

>Tc1-11_Xt(Froggy)

CAGTGGATATAAAAAGTCTACACACCCCTGGTAAAATGTCAGGTTCCTGTGCTGTACAAAAATGAGACAAAGATAAATCATTTCAGAACTTTTTCCACCTTTAATGTGACCTATAAACTGTACCACTCAATTGAAAAACAAACTGAAATCTTTTAGGTGGAGGGAAGAAAACCAAAAAAACTAAAATAATGTGGTTGCATAAGTGTGCACACCCTCTTCTAACTGGGGATGTAGCTGTGTTCAGAATTAAGCAATCACATTCAAAATCATGTTAAATAGGAGTCAGCATACACCTGCCATCATTTAAAGTGCCTCTGATTAACCCCAAATAAAGTTCAGCTGCTCTAGTTGGTCTTTCCTGACATTTTTTTAGTCGCATCCCACAGCAAAAGCCATGGTCCACAGAGAGCTTCCAAAGCATCAGAGGGATCTCATTGTTAAAAGATATCAGTCAGGAGAAGGGTACAAAAGAATTTCCAAGGCATTAGATATACCATGGAACACAGTGAAGACAGTCATCATCAAGTGGAGAAAATATGGCACAACAGTGACATTACCAAGAACTGGACGTCCCTCCAAAATTGATGAAAAGACGAGAAGAAAACTGGTCAGGGAGGCTACCAAGAGGCCTACAGCAACATTAAAGGAGCTGCAGGAATATCTGGCAAGTACTGGCTGTGTGGTACATGTGACAACAATCTCCCGTATTCTTCATATGTCTGGGCTATGGGGTAGAGTGGCAAGACGAAAGCCTTTTCTTACGAAGAAAAACATCCAAGCCAGGCTACATTTTGCAAAAACACATCTGAAGTCTCCCAAAAGCATGTGGGAAAAGGTGTTATGGTCTGATGAAACCAAGGTTGAACTTTTTGGCCATAATTCCAAAAAATATGTTTGGCGCAAAAACAATACTGCACATCACCAAAAGAACACCATACCCACAGTGAAGCATGGTGGTGGCAGCATCATGCTTTGGGGCTGTTTTTCTTCAGCTGGAACTGGGGCCTTAGTTAAGATAGAGGGAATTATGAACAGTTCCAAATACCAGTCAATATTGGCACAAAACCTTCAGGCTTCTGCTAGAAAGCTGAACATGAGGAGGAACTTCATCTTTCAGCATGACAACGACCCAAAGCATACATCCAAATCAACAAAGGAATGGCTTCACCGGAAGAAGATTAAAGTTTTGGAATGGCCCAGCCAGAGCCCAGACCTGAATCCGATTGAAAATCTGTGGGGTGATCTGAAGAGGGCTGTGCACAGGAGATGCCCTCGCAATCTGACAGATTTGGAGTGTTTCTGCAAAGAAGAGTGGGCAAATCTTGCAAAGTCAAAATGTGCCATGCTGATAGACTCATACCCAAAAAGACTGAGTGCTGTAATAAAATCAAAAGGTGCTTCAACAAAGTATTAGTTTAAGGGTGTGCACACTTATGCAACCACATTATTTTAGTTTTTTTGGTTTTCTTCCCTCCGCCTAAAAGATTTCAGTTTGTTTTTCAATTGAGTGGTACAGTTTATAGGTCACATTAAAGGTGGAAAAAGTTCTGAAATGATTTATCTTTGTCTCATTTTTGTACAGCACAGGAACCTGACATTTTACCAGGGGTGTGTAGACTTTTTATATCCACTG

>Tc1-12_Xt

CAGTGCTTTGCTAAAGTATTCACCCCCCTTGGCATTTTTCATGTTTTGTTACATTCCAACCTGTAATTTAAATGTTTCTTAATCTTATTTTATGTGATGGATCTGCACAAAATAGTCTAAGTTGGTGAAGTGAAATGAGAAAAATATATATAAAAAAGAATTTAAAAAAAATAAAAAACTGAAAATTGGCATGTGCATATGTATTCACCCCCTTTGCCATGAAGCCCCTAAAAAGTCCTGGTGCAACCAATTACCTTCAAAAGTCACATAATTAGTGAAATGAAGTCCACCTGTGTGCAATCTAAGTGTCACATGATCTGTCAGTATAAACACACCCTTTCTGAAAGGCCCCAGAGGCTTCAACACCACTAAGCAAGAGGCATCACACCATGAAGACCAAGGAGCTCTCCAAAGAAGTCAGGGACAAAGTTGTTGAGAAGTACAAGTCAGGGTTGGGTTATAAAAAAATATCCAAATCTTTGAGGATCCCCCGGAGCACCATCAAATCCATCATCTTCAAATGGAAAGAACATGGTACCACAACAAACCTGCCAAGAGAGGGCCGCCCACCAAAACTCACAGACCGGGCAAGGAGGGCATTAATCAGAGAGGCAGCACAGAGACCAAAGGTAACCCTGAAGGAGTTGCAGAGTTCCACAGCAGAGACTGGAGTATCTGTCCATAGGACCACAATAAGCCGTACACTCCATAGAGCTGGGCTTTATGGAAGAGTGGCCAGAAAAAAGCCATTACTTAACGTTAAAAATAAGAAGGCACGTTTTGAGTTTGCCAAAAGGCATGTGGGCGACTCCCCAAATGTATGGAGGAAGGTGCTCTGGTCAGATGAGACTAAAATTGAACTTTTCGGCCACCAAGGAAGATGCTATGTCTGGCGCAAACCCAACACATCCCATCACCCCAAGAACACCATCGCCACAGTGAAACATGGTGGTGGCAGCATCATGCTGTGGGGATGTTTTTCAGCAGCAGGGACTGGGAAACTGGTCCGAGTCGAGGGAAAGATGGATGGTGCTAAATACAGGGATATTCTTGAGCAAAACCTGTGTCAGTCTGCCTGTGATTTGAGACTGGGACGGAGGTTCACCTTCCAGCAGGACAATGACCCGAAGCATACTGCTAAAGCAACACTCGAGTGGTTTAAGGGGAAACATTTAAATGTGTTGGAATGGCCTAGTCAAAGTCCAGACCTCAATCCAATTGAGAATCTGTGGTCAGACTTGAAGATTGCTGTTCACAAGCGAAAACCATCCAACATGAAGGAGCTGGAGCAGTTTTGCCTTGAGGAATGGGCAAAAATCCCAGTGGCAAGATGTGGCAAGCTCATAGAGACTTATCCAAAGCGACTTGCAGCTGTAATTGCCGCAAAAGGTGGCTCTACAAAGTACTGACTTTAGGGGGGGTGAACAGTTATGCACGCTGAAGTTTTCTGTTATTTTGTCCTATTTGTTGTTTGCTTCACAATAAAAAAAAATACATCTTCAAAGTTGTAGGCATGTTCTGTAAATGAAATGGTGCAAACTCTCAAAACAATCCATTTTAATTCCAGGTTGTGAGGCAACAAAACATGAAAAATGCCAAGGGGGTGAATACTTTAGCAAAGCACTG

>Tc1-13_Xt

CAGTCATGTGAAAAAATTAGGACACCCTATGAAAGCCTGTGTGTTTTTGTAACATTCTTGGACATATGGATATTTAATCTCAATTTTAACAATACTGGGAGATTCAAGTAATATAACTAAACAATTAAAACTGAAGAAAAGACTTTTCAAAATCTTCTGTAAAATGTAATTCTACAAAAATGCAATTTCTGGTGAGGAATAAATCAGGACACCCCCACATTTAGTCCCACTTAAAATGGCTCAAATCACACACAGGTGTATCACATCAGGTGCACATGATTAGAACATCGTTACTCAGCATTTTGAAGGAGGTTTGCCCTATTTAAACCTCAGACATTTAGTTTGGTGTGCTCCTGACTGTTGAGGTGAGAGTGAACACCATGGTGAGATCAAAAGAGCTGTCTGAGGCCTTCAGAAAGAAGATTGTAGCAGCTTATAAGTCTGGTAAGGGATTTAAAAAGATCTCAAAAGAATTTGAAATCAGCCATTCCACTGTCCGGAAAATAGTCTACAAGTGGAGGACTTTCAAAACAACTGCCAACATGCCCAGGTCTGGCCGTCCAAGCAAGTTCACCCCGAGAGCAGACCGCAAGATGCTAAAAGAAGTCTCCAAAAACCCTAAAATGTCATCACGGGACCTACAGCAGGCTCTTGCTACTGTTGATGTGAAAGTGCATGCCTCTACAATCAGAAAGAGACTGCACAACTTTAACTTGCATGGGAGGTGTGCAAGGAGGAAACCTTTGCTCTCTAAGAGAAACATCAAGGCCAGACTGAAGTTTGCCAGAGAGAACGTAGACAAACACCAGGACTTCTGGAATAATGTTCTTTGGACAGATGAGTCTAAAATTGAATTATTTGGACACCAGAACAGAGGACATGTTTGGCGTAAACCAAATACAGCATTCCAGGAAAAGAACCTCATACCAACTGTGAAGCATGGAGGTGGAAGTGTCATGGTTTGGGGATGCTTTGCTGCAGCAGGACCTGGCCAGCTCACCATCATAGAATCCACCATGAATTCTACTGTGTATCAGAGGGTGCTTGAGGAACATGTGAGACCATCTGTAAGAAAATTAAAGCTGAAGCGGAACTGGACCCTGCAACACGACAATGACCCAAAACATACCAGTAAATCCACCAAGGACTGGCTGAAAACTAAGAAATGGAGAGTCCTGGAGTGGCCGAGTCAAAGCCCAGATCTTAATCCCATTGAGATGCTGTGGGGTGACTTGAAACGGGCTGTACATGCAAGAAACCCCTCAAACATCTCACAGCTGAAAGAATTCTGCATTGAGGAGTGGGGCAAACTTTCCTCAGACCGATGTCAGAGACTGGTAGATGGCTACAAGAAGCGTCTCACTGCAGTTATTTCAGCCAAAGGGGGTAACACTAGCTATTAGGGGGTAGGGTGTCCTAACTTTTTCCTCAGTTAGAATACACATTTTTGTTGATATCTTTTGTTTAATCAAAAGATCTTTTGAGTAAATCAAGGTTAATTTTTGTTGTTTACCTGCAATTAAATCCAGAGATAAATAAAAACAAGATTAGACATCGATATGTGAACATTTCTTAATAAAGAACTGAATATTTAATGGGGTGTCCTAATTTTTTCACATGACTG

>Tc1-14_Xt

CAGTGGTTCTCGAAAGTTTGTGAACCCTTTAAAATTTTCTATATTTTTATATGAATGTGACCTAAAACATCATCTGATTTTCAAACAAGTCCTAAAAGTAGATGAAGAAAACCTAGTTAAACAAATGAAACAAAAATTATTATATTTGGTCATGTATTTATTGAAAAAAAAATGATCCAATAACATATCTGCGTGTGGCAAAAGTAAGTGAATCCTTAGGATTATCATATAATTTGAAGGTGAAATCAGAGTCTGGTGTTTTCAGTCAATGGGATGACAATCAGGTGTGAGTGAGAGACCCTGTTTTATTTAAAGAACGGGGATCCAGCAAAGCCTGATCACACATACAACACATTTGTGGATGTGTATCATGGCTCGAACAAAGGAGGTGTCTGAGGACCTCAGAAAAAGAGTTGTTGATGCCCATAAAGCTGGAAAAGGTTACAAGACTATCTCTAAAGAGTTTGGACTCCACCAATCAACAGTCAGACAGATTGTGTACAAATGGAGGAAATTCAAGACCGTTGTTACCCTACCCAGAAGTGGTCGACCATCAAAGATAACTCCAACTGCAAGGCGTCTAATAGTCCGAGAGGTTACAAAGGAACCCAGGGTAACTTCTAAGCAACTGAAGGCCTGTCTCACATTGGCGAATGTTGATGTTCATGAGTCCACCATCAGGAGAACACTGAACAGCAATGGTGTGTATGGCAGGGTAGCAAGGAGAAAGCCACTGCTCTCCCCCAAAAATATTGCTGACCGTCTACAGTTTGCTAAAGATCATGTGGACAAACCAGAAGGATACTGGAAGAATGTTTTGTGGACGGATGAAGCCAAAATAGAACTTTTTGGCTTAAATGAGGAGCGTTACATTTGGAGAAAGAAAAACACTGCATTCCAGCATAAGAACCTTATCCCATCTGTGAAACATGGTGGTGGGAGTGTTCTGGTTTGGGCCTGTTTTGCTGCATCTGGGCCTGGACGGCTTGCCATCATTGATGGAACAATGAATTCTGAACTATACCAGAGAATTCTAAAGGAAAATGTCAAGACATCTGTCCGTGAACTGAATCTCAAGAGACAGTGGGTCATGCAGCAAGACAACGATCCTAAACACACAAGTCGTTCTACCAAAGAATGGTTAAAGAAGAATAAAGTGAATGTTCTGGAATGGCCAAGTCAAAGTCCTGACCTTAATCCAATTGAAATGTTGTGGAAAGACCTAAAGCGAGCGGTTCATGTGAGGAAACCCACCAACATCCAAGAGCTGAAGCTGTTCTGTATGGAGGAATGGGCTAAAATTCCTCCGAGTCGATGTGCAGGACTGATCAACAGTTACCGCAAACGTTTAGTTGCAGTTATTGCTGCACAAGGGGGTCACACCAAATACTGAGAGCACGATTCACTTACTTTTGCCACACGCAGATATGTTATTGGATCATTTTTTTTCAATAAATACATGACCAAATATAATAATTTTTGTTTCATTTGTTTAACTAGGTTTTCTTCATCTACTTTTAGGACTTGTTTGAAAATCAGATGATGTTTTAGGTCACATTCATATAAAAATATAGAANATTTTAAAGGGTTCACAAACTTTCAAGCACCACTG

>Tc1-15_Xt

CACTGCTCAAAAAAATAAAGGGAACACTTAAACAACACAATGTAACTCCAAGTCAATCACACTTCTGTGAAATCAAACTGTCCACTTAGGTAGCAACACTGATTGACAATCAATTTCACATGCTGTTGTGCAAATGGAATAGACAACAGGTGGAAATTATAGGCAATTAGCAAGACATCCCCAATAAAGGAGTGGTTCTGCAGGTGGTGACCACAGACCACTTCTCAGTTCCTATGCTTTCTGGCTGATGTTTTGGTCACTTTTGAATGCTGGCGGTGCTTTCACTCTAGTGGTAGCATGAGACGGAGTCTACAACCCACACAAATGGCTCAGGTAGTGCAGCTCATCCAGGATGGCACATCAATGCGAGCTGTGGCAAGAAGGTTTGCTGTGTCTGTCAGCGTAGTGTCCAGAGCATGGAGGCGCTACCAGGAGACAGGCCAGTACATCAGGAGACGTGGAGGAGGCCGTAGGAGGGCAACAACCCAGCAGCAGGACCGCTACCTCCGCCTTTGTGCAAGGAGGAACAGGAGGAGCACTGCCAGAGCCCTGCAAAATGACCTCCAGCGGGCCACAAATGTGCATGTGTCTGCTCAAACGGTCAGAAACAGACTCCATGAGGGTGGTATGAGGGCCCGACGTCCACAGGTGGGGGTTGTGCTTACAGCCCAACACCGTGCAGGACGTTTGGCATTTGCCAGAGAACACCAAGATTGGCAAATTCGCCACTGGCGCCCTGTGCTCTTCACAGATGAAAGCAGGTTCACACTGAGCACATGTGACAGACGTGACAGAGTCTGGAGACGCCGTGGAGAACGTTCTGCTGCCTGCAACATCCTCCAGCATGACCGGTTTGGCAGTGGGTCAGTAATGGTGTGGGGTGGCATTTCTTTGGGGGGCCGCACAGCCCTCCATGTGCTCGCCAGAGGTAGCCTGACTGCCATTAGGTACCGAGATGAGATCCTCAGACCCCTTGTGAGACCATATGCTGGTGCGGTTGGCCCTGGGTTCCTCCTAATGCAAGACAATGCTAGACCTCATGTGGCTGGAGTGTGTCAGCAGTTCCTGCAAGACGAAGGCATTGATGCTATGGACTGGCCCGCCCGTTCCCCAGACCTGAATCCAATTGAGCACATCTGGGACATCATGTCTCGCTCCATCCACCAACGCCACGTTGCACCACAGACTGTCCAGGAGTTGGTGGATGCTTTAGTCCAGGTCTGGGAGGAGATCCCTCAGGAGACCATCCGCCACCTCATCAGGAGCATGCCCAGGCGTTGTAGGGAGGTCATACAGGCACGTGGAGGCCACACACACTACTGAGCCTCATTTTGACTTGTTTTAAGGACATTACATCAAAGTTGGATCAGCCTGTAGTGTTTTTTTCCACTTTAATTTTGAGTGTGACTCCAAATCCAGACCTCCATGGGTTGATAAATTTGATTTCCATTGATAATTTTTGTGTGATTTTGTTGTCAGCACATTCAACTATGTAAAGAACGAAGTATTTAATAAGAATATTTCATTCATTCAGATCTAGGATGTCTTATTTTTGTGTTCCCTTTATTTTTTTGAGCAGTG

>Tc1-16_Xt(Jumpy)

CAGTCATGGCCAAAATTGTTGGCACCCCAGAAATTTTTCCAGAAAATCAAGTATTTCTCACAGAAAAGTATTGCAGTAACACATGTTTTGCTATACACATGTTTATTCCCTTTGTGTGTATTGGAACAGAACAAAAAAGGGAGGAAAAAAAGCAAATTGGACATAATGTCACACAAAACTCCAAAAATGGGCTGGACAAAATTATTGGCACCCTTTCAAAATTGTGGATAAATAAGATTGTTTCAAACATGTGATGCTCCTTTAAACTCACCTGGGGCAAGTAACAGGTGTGGGCAATATAAAAATCACACCTGAAAGCAGATAAAAAGGAGAGAAGTTCACTTAGTCTTTGCATTGTGTGTCTGTGTGTGCCACACTAAGCATGGACAACAGAAAGAGGAGAAGAGAACTGTCTGAGGACTTGAGAACCAAAATTGTGGAAAAATATCAACAATCTCAAGGTTACAAGTCCATCTCCAGAGATCTAGATTTGCCTTTGTCCACAGTGCGCAACATTATCAAGAAGTTTGCAACCCATGGCACTGTAGCTAATCTTCCTGGGCGTGGACGGAAGAGAAAAATTGATGAAAGGTTGCAACGCAGGATAGTCCGGATGGTGGATAAGCAGCCCCAAACAAGTTCCAAAGAAATTCAAGCTGTCCTGCAGGCTCAGGGAGCATCAGTGTCAGCGCGAACTATCCGTCGACATTTAAATGAAATGAAACGCTATGGCAGGAGACCCAGGAGGACCCCACTGCTGACACAGAGACATAAAAAAGCAAGACTACAGTTTGCCAAAATGTACTTGAGTAAGCCACAATCCTTCTGGGAAAACGTCTTGTGGACAGATGAGACCAAGATAGAGCTTTTTGGTAAAGCACATCATTCTACTGTTTACCGAAAACGGAATGAGGCCTACAAAGAAAAGAACACAGTACCTACAGTGAAATATGGTGGAGGTTCAATGATGTTTTGGGGTTGTTTTGCTGCCTCTGGCACTGGGTGCCTTGAATGTGTGCAAGGCATCATGAAATCTGAGGATTACCAAAGGATTTTGGGTCGCACTGTAGAGCCCAGTGTCAGAAAGCTGGGTTTGCGTCCGAGATCTTGGGTCTTCCAGCAGGACAATGACCCCAAACATACGTCAAAAAGCACCCAGAAATGGATGGCAACAAAGCGCTGGAGAGTTCTGAAGTGGCCAGCAATGAGTCCAGATCTAAATCCCATTGAACATCTGTGGAGAGATCTTAAAATTGCTGTTGGGAAAAGGCGCCCTTCCAATAAGAGAGACCTGGAGCAGTTTGCAAAGGAAGAGTGGTCCAAAATTCCCGGTGAGAGGTGTAAGAAGCTTATTGATGGTTATAGGAAGCGACTGATTTCAGTTATTTTTTCCAAAGGGTGTGCAACCAAATATTAAGTTAAGGGTGCCAATAATTTTGTCCAGCCCATTTTTGGAGTTTTGTGTGACATTATGTCCAATTTGCTTTTTTTCCTCCCTTTTTTGTTCTGTTCCAATACACACAAAGGGAATAAACATGTGTATAGCAAAACATGTGTTACTGCAATACTTTTCTGTGAGAAATACTTGATTTTCTGGAAAAATTTCTGGGGTGCCAACAATTTTGGCCATGACTG

>Maya

ATATACACTCACCAAAAGGATTATTAGGAACACCTGTTCAATTTCTCATTAATGCAATTATCTAATCAACCAATCACATGGCAGTTGCTTCAATGCATTTAGGGGTGTGGTCCTGGTCAAGACAATCTCCTGAACTCCAAACTGAATGTCAGAATGGGAAAGAAAGGTGATTTAAGCAATTTTGAGCGTGGCATGGTTGTTGGTGCCAGACGGGCCGGTCTGAGTATTTCACAATCTGCTCAGTTACTGGGATTTTCACGCACAACCATTTCTAGGGTTTACAAAGAATGGTGTGAAAAGGGAAAAACATCCAGTATGCGGCAGTCCTGTGGGCGAAAATGCCTTGTTGATGCTAGAGGTCAGAGGAGAATGGGCCGACTGATTCAAGCTGATAGAAGAGCAACTTTGACTGAAATAACCACTCGTTACAACCGAGGTATGCAGCAAAGCATTTGTGAAGCCACAACACGCACAACCTTGAGGCGGATGGGCTACAACAGCAGAAGACCCCACCGGGTACCACTCATCTCCACTACAAATAGGAAAAAGAGGCTACAATTTGCACAAGCTCACCAAAATTGGACAGTTGAAGACTGGAAAAATGTTGCCTGGTCTGATGAGTCTCGATTTCTGTTGAGACATTCAAATGGTAGAGTCAGAATTTGGCGTAAACAGAATGAGAACATGGATCCATCATGCCTTGTTACCACTGTGCAGGCTGGTGGTGGTGGTGTAATGGTGTGGGGGATGTTTTCTTGGCACACTTTAGGCCCCTTAGTGCCAATTGGGCATCGTTTAAATGCCACGGCCTACCTGAGCATTGTTTCTGACCATGTCCATCCCTTTATGACCACCATGTACCCATCCTCTGATGGCTACTTCCAGCAGGATAATGCACCATGTCACAAAGCTCGAATCATTTCAAATTGGTTTCTTGAACATGACAATGAGTTCACTGTACTAAAATGGCCCCCACAGTCACCAGATCTCAACCCAATAGAGCATCTTTGGGATGTGGTGGAACGGGAGCTTCGTGCCCTGGATGTGCATCCCACAAATCTCCATCAACTGCAAGATGCTATCCTATCAATATGGGCCAACATTTCTAAAGAATGCTTTCAGCACCTTGTTGAATCAATGCCACGTAGAATTAAGGCAGTTCTGAAGGCGAAAGGGGGTCAAACACCGTATTAGTATGGTGTTCCTAATAATCCTTTTGGTGTGTGTATAT

>DNA4_Xt

CAGGTATGGGATCCCTTATCCGGAAACCCGTTATCCAGAAAGTTCCGAATTACGGAAAGGCCATCTCCCATAGACTCCATTATAAGCAAATAATTCTAATTTTTAAAAATGATTTCCTTTTTCTCTGTAATAATAAAACAGTACCTTGTACTTGATCCCAACTAAGATATAATTAATCCTTATTGGAGGCAAAACAATCCTATTGGGTTTATTTAATGTTTAAATGATTTTTTAGCAGACTTAAGGTATGGAGATCCAAATTACGGAAAGATCCCTTATCCGGAAAACCCCAGGTCCCGAGCATTCTGGATAACAGGTCCCATACCTGTACAGGTATAGGACCCGTTATCCAGAATGCTCGGGACCAAGGGTATTCCGGATAAGGGGTCTTTCCGTAATTTGGATCTCCATACCTTAAGTCTACTAAAAAATCAATAAAACATTAATTAAACCCAATAGGATTGTTTTGCATCCAATAAGGATTAATTATATCTTAGTTGGGATCAATTACAAGGTACTGTTTTATTACTACAGAGAAAAAGGAAATCAGTTTTAAAATTCTGAATTATTTGATTAAAATGGAGTCTATGGGAGACGGGCTTTCCGTAATTCGGAGCTTTCTGGATAACGGGTTTCCGGATAAGGGATCCCATACCTG

>DNA5_Xt

CCGTATATACTCGAGTATAAGCCGATCCGAATATAAGCCGAGGTACCTAATTTTACCTAAGAAAACTGGAAAAACTTATTGACTCGAGTATAAGCCTAGGGTGGGAAATGCAGCAGCTACTGCTAAGTTTTAATAATCAAAATAAATACCAATAAAATTACATTAATTGAGGCATCAGTGGGGTATATGTTTTTCAATATTTATTTCAAAGAAAAACAGTAAACTAGCTCTGTAAGCGGAGAAGAGGGTCAACAAAAACAATATGAGTACTACCCCACGCTCATTGCACATTGGCAAACTGGCAGCAGACCCGGTCCCGGAGGAGATGTAAGGGGAAATAAGTATTGCTAGTGGGAGCCTAGGCCAGGGCACTGGAGGGTCTGGTTGCGGGTGGCCTAATTTGCACACAAAGGAGAGAGGGTGCTAGTCTAGAGGGACCCATGGCACCCGACTCGAGTATAAGCCGAGGGTGACTTTTTCAGCACATTTTGGGTGCTGAAAAACTAGGCTTATACTCGAGTATATACAG
