# Supplementary material for: Expression of Transposable Elements in Neural Tissues during Xenopus Development
Source: PLoS One. 2011 Jul 26;6(7):e22569. doi: 10.1371/journal.pone.0022569 (PMC3144230; doi:10.1371/journal.pone.0022569)
Supplement: File S2 — Protein sequences of Tc1-like elements of several species used in this study. (DOC) [file pone.0022569.s012.doc]

**File S2**

>Robertson.12_X.trop

GAMSAAGVGPLCFIKGRVNAASYQEILEHFMLPSAEKLYGNEDFIFQHDLAPAHSAKTTGKWFTAHGITVLNWPANS

>Robertson.19_X.trop

GAMSAAGVGPLCFIKGRVNAASYQEILEHFMLPSAEKLYGDEDFIFQNDLAPAHSAKTTGKWFTDHGITVLNWPANS

>Robertson.21_X.trop

GAMSAAGVGPLCFIKGRVNAASYQEILDHFMLPSAEKLYGDEDFIFQHDLAPAHSAKTTGKWFTDHGITVLNWPANS

>Robertson.22_X.trop

GAMSAAGVGPLCFIKGRVNAASYQEILEHFMLPSAEKLYGDEDFIFQHDLAPAHSAKTTGKWFTDHGITVLNWPANS

>Robertson.2_X.trop

G*FSSAGTGKLVRVNGKMDGAKYRAILEENLLESAKDLRLGRRFIPQQDNDPKHKARATMEWFKPKHIHVVEWPSQS

>Robertson.5m_X.trop

G*FAAPGPGRIAIIKGKMNF*VYQDILQENLRPSCP*LKLSRRWVLQQDNDPKHRSKSTTEWLKQKKIHLLDWPSQS

>TXr_X.laevis

MGLCEKVIAPLVKK*LNCGLSHLSSISKVYKAISKALGLQRTTVRAIIHKWQKHGTVVNLPRSGRPTKITP

RAQRQLIREATKDPRTTSKELQASLASIKVSVHDSTIRKRLGKNGLHGRFPRRKPLLSKKNIKARLNFAKKHLNDCQDFWENTLWTDETKVELFGRCASRYIWRKSNTAFQKKNIIPTVKYGGGSVMVWGCFAASGPGRLAVIDGTMNSTVYQKILKENVRPSVRQLKLKRSWVLQQDNDPKHTSKSTSEWLKKNKMKTLEWPSQSPDLNPIEMLWHDLKKAVHARKPSNKAELQQFCKDEWAKIPPERCKRLVASYRKRLIAVIAAKGGPTSY

>TXz_X.laevis

MRTKELSMQVKQVLLKLQKQKKPIREMATILGVAKSTVWYILRKKEITGELSNTKRPGRPRKTTVVDDHRIISMGKRNPFTRAKQVNNSLQEVGVSISKSTIKRRLYESKYRGGTARYKPLISIKNRKARLDFAKKHLKKPAQFWKNILWTDETKIKLYQNDGKSKKVWRRLWEQLMIQSIPQSSIKHLGGSVMAWGCMAASGTGTLVFIHHVTQDRSTRMNCELFRDTLSAQIQLNALKLIGRRFIMQMDNDPKHTAKATQEFIKAKKWNIAEYPSQSPDLNPIEHAFHLLKTKRRTERPTNKQQLKEAAAVKTWQSIKQEQTHNLLMSMSSTLQPVIAAKGFSTKY

>Tc1-12Xt_X.trop

MKTKEFSKEVRDKIVEXYKSGLGYKKISKTSRIPQST

IKSIIFKWKEHGTTTNLPREGRPPKLTDRARRALIRGAALRPKVTLKELQS

STAETGVSVHRTTIXAGLYGRVARKKPLRNVKNKKAPLEFAKRHVSDFPYV

WRKVLWSDETKILLFGHQGRCYIWRKPNTSHHPKNTMATVKHGGGNILLWG

CFSAAGTGKLVQVEGKMDGAKYXLEQNLCQSACDLRLGQRFTFQQDPKHAA

KATLKWYKGKHXNVLEWPSQSPDLNPIENMWSDMKIVVHEXKPSNVKELEQ

FCFEEWAKIPVARCGELIETYPKQLAADIAAKGGSKK

>Tc1-11Xt(froggy)_X.trop

RELPKHQRDLIVKRYQSGEGYKRISKALDIPWNTVKT

VIIKWRKYGTTVTLPRTGRPSKIDEKTRRKLVREATKSPTATLKEQQEYLA

STGCVVHVTTISRILHMSGLWGREARGKPFLTKKNIQARLHFAKTHLKSSK

SMWEKVLWSDETKVELFGHNSKKYVWRKNNTAHHQKNTIPTVKHGGGSIML

WGCFSSAGTGALAKIEGIMNSSKYQSILAQNLQASARKLNGRRNFIFQHDN

DPKHTSKSTKEWLHRKKIKVLEWSSQSPDLNPIENLWVDLKRAVHRRCPRN

LTDLECFCKEEWANLAKSKCAILIDPYPKRLSAVIKSKGASTK

>Tc1-10Xt(eagle)_X.trop

MKSKEHTRQVRDKVIEKFKAGLGYKKISKALNIPRST

VQAIIQKWKEYGTTVNLPRQGRPPKLTGRTRRALIRNAAKRPMVTLDELQR

STAQVGESVHRTTISRALHKVGLYGRVARRKPLLTENHKKSRLQFATSHVG

DTANMWKKVLWSDETKMELFGQNAKRYVWRKTNTAHHSEHTIPTVKYGGGS

IMLWGXCFSSAGTGKLVRVDGKMDGAKYRAILEENLLESAKDLRLGRRFTF

QQDNDPKHKARATMEWFKTKHIHVLEWPSQSPDLNPIENLWQDLKTAVHKR

CPSNLTELELFCKEEWARISVCRCAKLVETYPKRLAAVIAAKGGSTK

>Tc1-4Xt_X.trop

KTKELXTKDTRDKIVDLHKAGKGYGAIAKQLGENRST

VGAIVRKWKRLKTTVSLPRTGAPCKISPRGVSLMIRKVRNQPRTTREELVN

DMKRAGTTVSKVTVSRTLRRHGFKSCIAXRRXPLLKSSHVQARLKFANDHL

DDPEEAWEKVMWSDETKVELFGLNFTRRVWRKNKDELHPKNTIPTVKHGGG

NIMLWGCFSAKGTGRLHCIKERMNGAMYCEIFLSNNLLPSVRLKMGRGWVF

QHDNDPKHTARITKEWLRKRHIKVLEWPSQSPDLNPIENLWRELKLCVAQR

QPRNLTDLEEICVEEWAKIPVAVCANLVKNYRKRLTSVIANKGFCTK

>Quetzal_A.albimanus

MTREELSVSKRQDIIRLHGAQGKSYTEIAMLTNINRNTVARVIQRYKYEGRVSNLPRKGRPSVCTDRMRR

AIKRLVDAEPEISAQSVAIVLNERHGIAISCETVRRYIHKFGYKAYNRRKKPQISPINRKRRLEFAKKYV

NHPPEFWKKVLFTDESKFNIFGWDGTIKVWRPPGEGLNPKYTAKTVKHNGGGVLVWGCMAANGVGNLQVI

DGIMDQYVYINILKQNLGPSLEKLGMSQDYWFQQDNDPKHTAFNSRLFLLYNTPHQLKSPPQSPDLNPIE

HAWELLERKIRQTRIKNRVDLENKLKEAWITISEDYTQNLVNSMPRRLAEVIKMKGYATRY

>Tcb1_C.briggsae

MDRNILRACREDPRRTSTDIQLSVTSPNEPVPSRRTIRRRLQVAGLHGRRPVKKPLVSLKNRKARVEWAK

QHLSWGPREWANHIWSDESKFNMFGTDGIQWIRRPIGSRYAPQYQCPTVKHGGGSVMVWGCFSDTSMGPL

KRIVGTMDRYVYEDILENTMRPWARANLGRSWVFQQDNDPKHTSGHVANWFRRRRVNLLEWPSQSPDLNP

IEHMWEELERRLKGVRASNANQKFAQLEAAWKSIPMTVVQTLLESMPRRCKAVIDAKGYPTKY

>Tcb2_C.briggsae

MDRNIVRVSREDPQRSSTDIQMVVKTPNEVTPSLRTVRRRLQDAGLHGRRPAKKPSISKKNRIARVAWAR

AHLHWGRQDWANHVFSDESKFNLFGTDGIKWIRRPVGCRFDPSYQLQTVKHGGGSVMVWGCFSGTSMDPL

RRIDSIMDRFVYEDILENTMRPWARSTVGRAFVFQQDNDPKHTSKHIKEWFRRRHVDLLDWPSQSPDLNP

IEHLWEHVERHVRGVRASNANQKFDQLQDVWQAIPMSVIDTILDSMPRRCQTVIDAKGFPTKY

>Tc1_C.elegans

MDRNILRSAREDPHRTATDIQMIISSPNEPVPSKRTVRRRLQQAGLHGRKPVKKPFISKK

NRMARVAWAKAHLRWGRQEWAKHIWSDESKFNLFGSDGNSWVRRPVGSRYSPKYQCPTVK

HGGGSVMVWGCFTSTSMGPLRRIQSIMDRFQYENIFETTMRPWALQNVGRGFVFQQDNDP

KHTSLHVRSWFQRRHVHLLDWPSQSPDLNPIEHLWEELERRLGGIRASNADAKFNQLENA

WKAIPMSVIHKLIDSMPRRCQAVIDANGYATKY

>Uhu_D.heteroneura

MVNLSTTTVFNIIRRFVDENRIEDKGRKAPNKIFTEQEERRIIRKIRENPKLSAPKLTQQVQDEMGKKCS

VQTVRRVLHNHDFNARVPRKKPFISTKNKGTRMTFAKTHLDKDLEFWNTIIFEDESKFIIFGSDGRNYVR

RQSNTELNPKNLKATVKHGGGSVMVWACISAASVGNLVCIETTTDRNVDLSI

>Minos_D.hydei

MVRGKPISKEIRVLIRDYFKSGKTLTEISKQLNLPKSSVHGVIQIFKKNGNIENNIANRGRTSAITPRDK

RQLAKIVKADRRQSLRNLASKWSQTIGKTVKREWTRQQLKSIGYGFYKAKEKPLLTLRQKKKRLQWARER

MSWTQRQWDTIIFSDEAKFDVSVGDTRKRVIRKRSETYHKDCLKRTTKFPASTMVWGCMSAKGLGKLHFI

EGTVNAEKYINILQDSLLPSIPKLSDCGEFTFQQDGASSHTAKRTKNWLQYNQMEVLDWPSNSPDLSPIE

NIWWLMKNQLRNEPQRNISDLKIKLQEMWDSISQEHCKNLLSSMPKRVKCVMQAKGDVTQF

>S-element_D.melanogaster

MPGKRLAFEVTQLIYYNHQLGKSIPELVEIFSVSRKTVYNILNRXXKEGRLEPKSGGGCKTKINKRVDRL

IMRKAIANPRISVRSLAQDIREECHLTVSHETVRQVILRHRYSSRVARKKPLLSEINIEKRHSFAVSMMD

HAEEYWDDVIFCDETKMMLFYNDGPSRVWRKPLSALETQNIIPTIKFGKLSVMIWGCISSHGVGKLAFIE

STMNAVQYLDILKTNLKASAEKFGLFSNNKPNFKFYQDNDPKHKEYNVRNWLLYNCGKVIDTPPQSPDLN

PIENLWAYLKKKVAKRGPKTRQQLMAAIIEEWEKIPLEYDLKKLIHSMKKRLQLVAKANGGHTKY

>Bari_D.melanogaster

MPKTKELTVEARAGIVARFKAGTPAAKIAEIYQISRRTVYYLIKKFDTVGTLKNKKRSGRKPVLDQRQCR

QILGVVAKNPSASPVKIALESKNTIGKQVSSSTIRRRLKEADFKTYVVRKTIEITPTNKTKRLRFALEYV

KKPLDFWFNILWTDESAFQYQGSYSKHFMHLKNNQKHLAAQPTNRFGGGTVMFWGCLSYYGFGDLVPIEG

TLNQNGYLLILNNHAFTSGNRLFPTTEWILQQDNAPCHKGRIPTKFLNDLNLAVLPWPPQSPDLNIIENV

WAFIKNQRTIDKNRKREGAIIEIAEIWSKLTLEFAQTLVRSIPKRLQAVIDAKGGVTKY

>HB1_D.melanogaster

MLILKLRKEGKTYKDIQKTLKCSAKMVSNAIKYKWKPENRGTKHKTTDIEDRRIVSYSKV

YRFASFRDIKSELNLGISDVTIRRRLLNQNFSARSPRKVPLPSPRHIKARLSLAKTYLNW

PVSKWRNILWTDGSKIMLFGGTGSLQYI

>Tzf_D.rerio

MSKHKPSMKTKELSVDLRDRIVSRHKAGEGYRNISAALKVPMSTATSIIRKWKMFGTTRTRPRAGRPSKLSVRGRRSLVREVINNPMVTLSELQRSSVERGEPYRRTTICAAIHQSGLYGRVARRKATPHLEFAKRHLKDSQNIRNKILWSDETKIELFGVNARRYVWRKPGSAHHQANTIPTVKHGGGSIMLWGCFSAAGTGRLVRIEGKMNAAMYRDILNKDLLQSALDLRLGRRFIFQQDNDPKHTAKISMEWLHNNSVNVLEWPSQSPDLNPIEHLWRDLKMAVHRRFPSNLIELERYCKEEWAKIPKDRCAKLVASYSKRLEAVIAAKGASTKY

>Tdr1a_D.rerio

MGKSKEXSQDIRKRIVDLHKSGSSLVTISRCLKVPHSSVQTNLRNYKQHGNVQPSYRSGRRRILCPRDERAMVRTVCINPRTKAKDLVKMLAEAGKRVSLSTVKRVLYXHGLKGHSARKKPLLQKHHKKARLQFAKLHWEKDLCFWRHVLWSDGTKIELFGHNDQRYIWRTKGKAHKPKNTIPTVQYGGGSIMLWGCFAAGGTGPLHSIDGIMRKEHYVEILKQHLKYSARKLKLGHKWVFQMDHDPKHTAKLVKMCFKDNRVNVLEWPSQSPDLNPIENLWAELKKLVRARQPTNLTQLHQFCQEEWAKIPANYCEKLVEGYPKHLTKVIQFKSKATKY

>Paris_D.virilis

MPGQRINENVIQLVYFHYHKGKCAKELAEMFSIKLRTIYNIINRAEKENRLELKHSGGRPAKLSRRDHSK

ILKQINENPQTSLRQLALDLKNDCNKTVSHETVRKVLKMHKYSSQIARKKPLLSAVNIQRRLNFSITNVN

KPAEYWDDVIFCDETKIMLYYHDGPSKVWRKPNTALEQKNIIPTVKFGKLSVMVWGCISSKGVGELRIFN

DVMTKEFYLDILKNELSRSAIKFGFVDPQNPSKQRYKLYQDNDPKHKSFLCRTWLLYNCSKVIDTPAQSP

DLNPIENLWAFLKKRVGKRSPTNKNALIKAIQEEWIKIPEIYDLHNLIQSMSRRLRAVMDANGQYTKY

>Impala_F.oxysporum

MPRGKELTPSLRSRICELKKQGYSYSQIHKHFPYIPLGTIKTTCRREAQRGAENTTLPRSGAPRKLTEEQ

RDQIYDTVITDPHVTTRDLLDSVDNVIKKRSLRYLLREMNKRKWIQKKRVALTPLQARKRLDWAIRYQGI

DWRRVKWSDECMVRRGQGMRPIWTFLSPREALRVQDVQEARRLGAVRQMFWAAFGHRSRTPLVPLVGNVN

AIGIYELYSFILPWFLQSGDIFMHDNASVHTARIVKALLEELGVDLMTWPPYSPDLNPIENLWALMKAEI

YRLHPELTHTEDTVATQHALVLAAMEAWDNIEDRVLKNLCETMPNRVTAVITAEGWYTKY

>Tes1_E.stouti

MGSQLQLFCNDGSNFSLGSCKQFLHVSQLLLISYKPSVCKKAVLEQIVLLHPLKHYLDNIALQEVVYCYH

NGEKNTINKGRQRDHYNPSKCRSFLWRNCKEAKVSASTVSYTIKRHLETAGNSDRKRSGRPKATTQSEDE

CLRFNQKTIRRWQNKKKRFAWAMKHRQWTTENWKKALWTDESKFEIFVSSRRVFVRCVGKRIVPHCVTPT

VKHGGGSLMIWGSFAGSRVGDLHRVTGTLNRKGYHSILQRHAIPSGLRLVGQGFILQQDNDPKHTSRLCQ

NDLRREEQDGRLQIMEWPAQSPDLNPIELVWDELDRRVKAKQPTSATHLWELLQQSREELSEQYLISIVE

RMPVCSAVISAKGGYFDESKISIKFCSTKRFHDFFLNLQLLVCSMLLFQSIPKH

>Tc1_H.contortus

MARHTGIRNLRQDQVDAIIRSFHAGLTSRQVSEIQGVTIRCVQRIWKKYKDTGSVEVKKHPGAARTTSRL

VDRNIVRLARNDPRLTAAEILREISTPEGSNLSLSTVQRRLREAGLFGRRPAKKPLISAKNRKARLDWAQ

AHKNWTVRQWRKVIWSDESKFLLFGTDGIKFVRRPVGTRYHPSYQLPTVKGGGDSVMVHGSFCGKGAGPL

HRIEGKMDAKMYLNIMETVIWPFVRSTARRGFIFQQDNDPKHKSKLLTKWFRDNNVPLLMWPSLSPDLNA

TENLWERLKHQVKGLRARNEHEKFNQLKTAWENIPQEEIDKLIESMPCRCQAVIDARGHATKY

>PPTN_P.platessa

MKTKELTKQVRDKVVEKYEAGLGYKKISRALNISLSTIKSIIRKWKEYGTTANLPRGGRPPKLKSRTRRK

LIREATRRPMVTLEELQRSTAEVGESVHRTTISRLLHKSGLYGRVARRKPLLKGIHKKSRLEFARSHVGD

TANMWKKVLWSDETKIELFGLNAKRYVWRKPNTAHHPEHTIPTVKHGGGSIMLWGCFSSAGTGKLVRIEG

KMDGAKYREILEENLMQSAKDLRLGRRFIFQQDNDPKHTARATKEWFGLKNVNVLKWPSQSPDLNPIENL

WQDLKIAVHRRSPSNLTELHLFCQEEWTNLSISRCAKLVETYPKRLAAVIAAKGGSTKY

>RTTN_R.temporaria

MKTKELSKQVRDNVVEKYKSGLGYKKISKSLMIPRSTIKSIITKWKEHGTTANLPRDGRTPKLTDRARRALIREAAQRPKVTLEELQSSTAETGVSVHRTTISRTLHRVGLYGRVARRKPLLSAKNKMARFEFAKRHVGDSQNVWRKVLWSDETKI*LFGHQRKRYVWRKPNTSHHPKNTIPTVKHGGGSIMLWGCFSAVGTGKLVRVEGKMDGAKYRDILEQNLYHSVCDLRLGWRFTFQQDNDPKHTAKATLEWFKGKHVNVLEWPSQSPDLNPIENLWSDLKIAVHKCKPSNLKELEQFCKEEWAKIPVVRCGKLIETYPKRLGAVIAAKGGSTKY

>Frog_prince_R.pipiens

MPRPKEIQEQLRKKVIEIYQSGKGYKAISKALGIQRTTVRAIIHKWRRHGTVVNLPRSGRPPKITPRAQR

RLIQEVTKDPTTTSKELQASLASVKVSVHASTIRKRLGKNGLHGRVPRRKPLLSKKNIKARLNFSTTHLD

DPQDFWDNILWTDETKVELFGRCVSKYIWRRRNTAFHKKNIIPTVKYGGGSVMVWGCFAASGPGRLAVIK

GTMNSAVYQEILKENVRPSVRVLKLKRTWVLQQDNDPKHTSKSTTEWLKKNKMKTLEWPSQSPDLNPIEM

LWYDLKKAVHARKPSNVTELGQFCKDEWAKIPPGRCKSLIARYRKRLVAVVAAKGGPTSY

>SSTN_S.salar

MKTKELSKQVRDKVVEKYRSGLGYKKISETLNIPRSTIKSIIKKLKEYGTTTNLPREGRPPKLTDQARRALIREATKRPKITLKELQSSTAEIGVSVHRTTLSRTLHRAGLYGRVARKKPLLKEKNKQTRLVFAKRHVGDSPNIWKKVLWSDETKIELFGHQGKRYVWRKPNTSHHPENTIPTVKHGGGSIMLWGCFSSAGTGKLVRIEGMMDGAKYREILEGNLFQSSRDLRLGRRFTFQQDNDPKHTAKATLEWFKGKHLNVLEWPSQSPDLNPIENLWYDLKIAVHQRNPSNLKELEQFCLEEWAKIPVARCAKLIETYPKRLAAVIAAKGGSTKY

>DTSsa1_S.salar

MAETKELSKDVRDKIVDLHKAGMGYKTIAKQLGEKVTTVGVIIRKWKKHKRTVNLPRPGAPCKISPRGVA

MIMRTVRNQPRTTWEDLVNDLNAAGTIVTKKTIGNTLRREGLKSCSARKVPLLKKAHIHARLKFASEHLN

DSEDNWVKVLLADETKMELFGINSTHRVWRRRNAAYDPKNTIPTVKHGGGNIMLWGCFSAKGTGQLHCIK

GTMDGAMYRQILGENLLPSARALKMGHGCVFQHDNDPKHTAKATKEWLKKKHIKVLEWPSQSPDLNPIEN

LWRELKVRVAKRPPRNLNDLEKICKEEWDKIPPEMCANLVANYKKCLTSVIANKGFSTKY

>DTSsa2_S.salar

MRAVARRFAVSVSVVSRAWRRYQETGQYIRRCGGGRRRATTQQQDRYLRLCARRSRRSTARALQNDLQQA

TNVHVSAQTVRNRLHDGGMRARRPQVGVVLTAQHRAGRLAFAREHQDWQIRHWRPVLFTDERRFTLSTCD

RRDRVWRCCGECFAACNILQHDRFGGGSVMVWGGISLGGRTALHVLARGSLTAIRYRDEILRPLVRPYAG

AVGPGFLLMQYNARPHVAGVCQQFLQEEGIDAMDWPARSPDLNPIEHIWDIMSRSIHQRHVAPQTVQELA

DALVQVWEEIPQETIRHIIRSMPRRCREVIQARGGHTHY

>Salt-1_S.salar

MRLEIELWCILPPLIILEMLLQIKVHLR*IQLIGHDLERHTPVYIRSHS*QCMSEQKPSHEVDGIVXRAPRQDCL*AQI*GRVPKKL*KTPRTQWPPSFFNERSLEQPKLFIELTAWPN*AIGGEWP*SGR*PRNPMVTLTELQSSSVRIGEPSRRTTISAALHKSGLYGRVARRKPLLSKREMTACLGFAKRHLKDSQIM*NKILWSDETNIELPGLNAKHHVNRKPGTIPTVKKGGDSIMLWLCFSAAGTG*LARIEGKMNGAKYREILDENLLQSTQDLRLGRRRFTFQQDNNPKHTAKTT*EWLRDKSLNVLEWPSQSPDLKPIKLWRDLKISVQQRSPSWLTELERICVEEWEKLP*YSCAKLVVS*PRTGVIAAKGASTKY

>Tss_S.salar

MGKSKEISQDLRKKIVDLHKSGSSLGASSKRLKVPRSSVQTIVRKYKHHGTTQPSYRSGRRXVLSPRDERTLVRKVQINPRTTAKDLVKMLEETGTKASISTVKRVLYRHNLKGRSARKKPLLQNRHKKARLRFATAHGDKDCTFWRNVLWSDETKIELFGHNDHRYVWRKKGEACKPKNTIPNVKHGGGSIMLXGCFAAGGTGALHKIDGIMRXENXVDILKQHLKTSVRKLKLGRKWVFQMDNDPKHTSKVVAKWLKDNKVKVLEWPSQSPDINPIEHLWAELKKCVRARRPTNLTQLHQLCQEEWAKIXPTYCGKLVEGYPKHLNQLKFKGNATKY

>Put_transposasa_XP_001338227.1_D.rerio

MGKTADLTAQARGRVQEMGYRYHIPQVKPLLNQKQRQKRARVWRKTGEREMPKCLKSSVKYPQSGMVWGA

MSAADVGPLCFIKGRVNAASYQKILEHFMLPSTKKLYGDEDFIFQQDLAPAHSAKTTGKWFTDHGITVLN

WPANSPDLNPIENLRDIVKRKLRDARPNALDELKAAIEASWASITPQQCHRLIASMPHRIEAVISAKGFP

IKY

>Put_transposasa_XP_001345300.1_D.rerio

MRKGDRTLRKIVEKDRFQTLGDLRKQWTESGLETSKATVYRRVQEMGYRCRIPQVKPLLNQKQQQKRLTW

AAEMQHWTVAQWSKVLFSDEIQFCMSFGNQGARVWRKTGEMEMPKCLKSSVKYPQTVMVWGAMSAAGVGP

LCFIEGRVNEASYQDILEHFFMDMQTGKWFTDHGITVLNWPANSYDLNPIENMWDLVKRKLRDARPNTLD

ELKTPQQCHRLITSMPRHIEAVISARILGQVLSA

>Sleeping_Beauty

MGKSKEISQDLRKKIVDLHKSGSSLGAISKRLKVPRSSVQTIVRKYKHHGTTQPSYRSGRRRVLSPRDER

TLVRKVQINPRTTAKDLVKMLEETGTKVSISTVKRVLYRHNLKGRSARKKPLLQNRHKKARLRFATAHGD

KDRTFWRNVLWSDETKIELFGHNDHRYVWRKKGEACKPKNTIPTVKHGGGSIMLWCGFAAGGTGALHKID

GIMRKENYVDILKQHLKTSVRKLKLGRKWVFQMDNDPKHTSKVVAKWLKDNKVKVLEWPSQSPDLNPIEN

LWAELKKRVRARRPTNLTQLHQLCQEEWAKIHPTYCGKLVEGYPKRLTQVKQFKGNATKY

>Tc1_R.catesbiana

MGKTADLTVVQKTVIDTLHKEGKSQKVIAKEAGCSQSAVSKYINGKLSGRKKCGRKRCTSNRDNCNLEKI

VKQRPFKNLGEIHKEWTTAGVSASRATTHRRIQDMGYSCRISCVKPLLNQRQRHKRLTWAKGKKDWTVAQ

WSKVLFSDESKFCISFGNQGPRVWRKSGEAQNPSCMRSSVKFPQSVMIWGAMSSAGVGPLCFIKSKVSAA

LYQEILEHYMLPSAVQLYGDADFIFQQELAPAHTAKSINTWFNDHGITVLDWPANSSDLNPIENMWGIVK

RKMRDTRPNNEDELRATIKAAWASITPQQCHRLIASMPQRIDAVIHAKGALTKY

>Tc1-2_Xt(this work)

MGKTADLTAVQKAIIDTLKQEGKTQKEISERIGCSQSAVSRHLSGKSVGRKKCGRKRCTTRRG DRALRKIVEKDRFQTLGDLRKQWTESGVETSRATMYRRVQEMGYRCRIPQVKPLLNQKQR QKRLTWATEKQHWTVAQWSKLLFSDESKFCMSFRNQGARVWRKTGEREMPKCLKSSVKYP QSVMVWGAMSAAGVGPLCFIKGRVNAASYQEILEHFMLPSAEKLYGDEDFIFQHDLAPAH SAKTTGKWFTDHGITVLNWPANSPDLNPIENLWDIVKRKLRDTRPNSLDELKAAIEASWA SITPQQCHRLIASMPRRIEAVISAKGFPTKY
